# Supplementary material for: The spatiotemporal evolution and driving factors of urban–rural integration over the past eight years in China: Evidence from 31 provinces
Source: PLoS One. 2025 Dec 2;20(12):e0337317. doi: 10.1371/journal.pone.0337317 (PMC12671771; doi:10.1371/journal.pone.0337317)
Supplement: Attachment — Submitted filename: PONE-D-25-17463_reviewer.pdf [file pone.0337317.s003.pdf]

PLOS ONE

The Spatiotemporal Evolution and Driving Factors of Urban–Rural Integration Over the Past Seven Years in China: Evidence from 31 Provinces

--Manuscript Draft--

**Manuscript Number:**	PONE-D-25-17463
**Article Type:**	Research Article
**Full Title:**	The Spatiotemporal Evolution and Driving Factors of Urban–Rural Integration Over the Past Seven Years in China: Evidence from 31 Provinces
**Short Title:**	The Spatiotemporal Evolution and Driving Factors of Urban–Rural Integration Over the Past Seven Years in China
**Corresponding Author:**	Wei Wang<br>Hunan Agricultural University<br>CHINA
**Keywords:**	urban-rural integration; entropy weighting method; coupling coordination degree model; geographic detector model
**Abstract:**	Urban-rural integration development is a key pathway to breaking down the inherent barriers between urban and rural areas and achieving coordinated regional development. This paper uses panel data, along with methods such as the entropy method, coupling coordination degree model, and geographical detector model, to deeply analyze the spatial-temporal evolution characteristics and main influencing factors of urban-rural integration development in 31 provinces, autonomous regions, and municipalities in China from 2015 to 2022. The research results show that, from a temporal perspective, the overall urban-rural integration development in China has shown an upward trend, but the growth rate is uneven, specifically exhibiting a development pattern of "rapid growth—slow development—fluctuating rise." From a spatial perspective, urban-rural integration development shows obvious regional disparities, with the eastern region taking the lead in development, the central region quickly rising, and the western region showing broad development prospects. In addition, the geographical proximity effect plays an important role in shaping the spatial dynamics of urban-rural integration development. As for the main influencing factors, broadband internet access, per capita disposable income, education expenditure, unemployment insurance coverage, and green coverage rate have become the core drivers of urban-rural integration development. Based on these findings, this paper suggests that the government should formulate differentiated development strategies based on different development stages, strengthen coordination and cooperation between regions, and adopt systematic development strategies to effectively address various challenges, thereby accelerating the process of urban-rural integration development.
**Order of Authors:**	Hui Liu
	Wei Wang
**Additional Information:**	
**Question**	**Response**
**Financial Disclosure**<br><br>Enter a financial disclosure statement that describes the sources of funding for the work included in this submission. Review the submission guidelines for detailed requirements. View published research articles from *PLOS ONE* for specific examples.	Yes

Powered by Editorial Manager® and ProduXion Manager® from Aries Systems Corporation

<table>
<tr><td>This statement is required for submission and will appear in the published article if the submission is accepted. Please make sure it is accurate.

Funded studies
Enter a statement with the following details:
• Initials of the authors who received each award
• Grant numbers awarded to each author
• The full name of each funder
• URL of each funder website
• Did the sponsors or funders play any role in the study design, data collection and analysis, decision to publish, or preparation of the manuscript?

Did you receive funding for this work?</td><td></td></tr>
<tr><td>Please add funding details.
as follow-up to "Financial Disclosure

Enter a financial disclosure statement that describes the sources of funding for the work included in this submission. Review the submission guidelines for detailed requirements. View published research articles from PLOS ONE for specific examples.

This statement is required for submission and will appear in the published article if the submission is accepted. Please make sure it is accurate.

Funded studies
Enter a statement with the following details:
• Initials of the authors who received each award
• Grant numbers awarded to each author
• The full name of each funder
• URL of each funder website
• Did the sponsors or funders play any role in the study design, data collection and analysis, decision to publish, or preparation of the manuscript?</td><td>This research was funded by National Social Science Late Grant Program, grant number 22FGLB007 and Project of Natural Science Foundation of Hunan Province, grant number 2023JJ30313.</td></tr>
</table>

Did you receive funding for this work?"	
Please select the country of your main research funder (please select carefully as in some cases this is used in fee calculation).<br>as follow-up to "**Financial Disclosure**<br><br>Enter a financial disclosure statement that describes the sources of funding for the work included in this submission. Review the submission guidelines for detailed requirements. View published research articles from *PLOS ONE* for specific examples.<br><br>This statement is required for submission and **will appear in the published article** if the submission is accepted. Please make sure it is accurate.<br><br>**Funded studies**<br>Enter a statement with the following details:<br>• Initials of the authors who received each award<br>• Grant numbers awarded to each author<br>• The full name of each funder<br>• URL of each funder website<br>• Did the sponsors or funders play any role in the study design, data collection and analysis, decision to publish, or preparation of the manuscript?<br><br>Did you receive funding for this work?"	CHINA - CN
**Competing Interests**<br><br>Use the instructions below to enter a competing interest statement for this submission. On behalf of all authors, disclose any competing interests that could be perceived to bias this work—acknowledging all financial support and any other relevant financial or non-financial competing interests.<br><br>This statement is required for submission and **will appear in the published article** if	The authors have declared that no competing interests exist.

the submission is accepted. Please make sure it is accurate and that any funding sources listed in your Funding Information later in the submission form are also declared in your Financial Disclosure statement.<br><br>View published research articles from *PLOS ONE* for specific examples.<br><br>**NO authors have competing interests**<br><br>Enter: *The authors have declared that no competing interests exist.*<br><br>**Authors with competing interests**<br><br>Enter competing interest details beginning with this statement:<br><br>*I have read the journal's policy and the authors of this manuscript have the following competing interests: [insert competing interests here]*<br><br>* typeset	
**Ethics Statement**<br><br>Enter an ethics statement for this submission. This statement is required if the study involved:<br><br>• Human participants<br>• Human specimens or tissue<br>• Vertebrate animals or cephalopods<br>• Vertebrate embryos or tissues<br>• Field research<br><br>Write "N/A" if the submission does not require an ethics statement.<br><br>General guidance is provided below. Consult the submission guidelines for detailed instructions. **Make sure that all information entered here is included in the Methods section of the manuscript.**	N/A

**Format for specific study types**<br><br>**Human Subject Research (involving human participants and/or tissue)**<br>• Give the name of the institutional review board or ethics committee that approved the study<br>• Include the approval number and/or a statement indicating approval of this research<br>• Indicate the form of consent obtained (written/oral) or the reason that consent was not obtained (e.g. the data were analyzed anonymously)<br><br>**Animal Research (involving vertebrate animals, embryos or tissues)**<br>• Provide the name of the Institutional Animal Care and Use Committee (IACUC) or other relevant ethics board that reviewed the study protocol, and indicate whether they approved this research or granted a formal waiver of ethical approval<br>• Include an approval number if one was obtained<br>• If the study involved *non-human primates*, add *additional details* about animal welfare and steps taken to ameliorate suffering<br>• If anesthesia, euthanasia, or any kind of animal sacrifice is part of the study, include briefly which substances and/or methods were applied<br><br>**Field Research**<br><br>Include the following details if this study involves the collection of plant, animal, or other materials from a natural setting:<br>• Field permit number<br>• Name of the institution or relevant body that granted permission	
**Data Availability**<br><br>Authors are required to make all data underlying the findings described fully available, without restriction, and from the time of publication. PLOS allows rare exceptions to address legal and ethical concerns. See the PLOS Data Policy and FAQ for detailed information.	Yes - all data are fully available without restriction

A Data Availability Statement describing where the data can be found is required at submission. Your answers to this question constitute the Data Availability Statement and **will be published in the article**, if accepted.<br><br>**Important:** Stating 'data available on request from the author' is not sufficient. If your data are only available upon request, select 'No' for the first question and explain your exceptional situation in the text box.<br><br>Do the authors confirm that all data underlying the findings described in their manuscript are fully available without restriction?	
**Describe where the data may be found in full sentences. If you are copying our sample text, replace any instances of XXX with the appropriate details.**<br><br>• If the data are **held or will be held in a public repository**, include URLs, accession numbers or DOIs. If this information will only be available after acceptance, indicate this by ticking the box below. For example: *All XXX files are available from the XXX database (accession number(s) XXX, XXX.).*<br>• If the data are all contained **within the manuscript and/or Supporting Information files**, enter the following: *All relevant data are within the manuscript and its Supporting Information files.*<br>• If neither of these applies but you are able to provide **details of access elsewhere**, with or without limitations, please do so. For example:<br><br>*Data cannot be shared publicly because of [XXX]. Data are available from the XXX Institutional Data Access / Ethics Committee (contact via XXX) for researchers who meet the criteria for access to confidential data.*<br><br>*The data underlying the results presented in the study are available from (include the name of the third party*	All relevant data are within the manuscript and its Supporting Information files.

Powered by Editorial Manager® and ProduXion Manager® from Aries Systems Corporation

and contact information or URL).<br>• This text is appropriate if the data are owned by a third party and authors do not have permission to share the data.<br><br>* typeset	
Additional data availability information:	

Powered by Editorial Manager® and ProduXion Manager® from Aries Systems Corporation

Manuscript Click here to access/download;Manuscript;Manuscript.docx

The Spatiotemporal Evolution and Driving Factors of Urban–Rural Integration Over the Past Seven Years in China: Evidence from 31 Provinces

Hui Liu and Wei Wang*

College of Public Administration and Law, Hunan Agricultural University, Changsha, China;

* Correspondence: weiw@hunau.edu.cn

Abstract: Urban-rural integration development is a key pathway to breaking down the inherent barriers between urban and rural areas and achieving coordinated regional development. This paper uses panel data, along with methods such as the entropy method, coupling coordination degree model, and geographical detector model, to deeply analyze the spatial-temporal evolution characteristics and main influencing factors of urban-rural integration development in 31 provinces, autonomous regions, and municipalities in China from 2015 to 2022. The research results show that, from a temporal perspective, the overall urban-rural integration development in China has shown an upward trend, but the growth rate is uneven, specifically exhibiting a development pattern of "rapid growth—slow development—fluctuating rise." From a spatial perspective, urban-rural integration development shows obvious regional disparities, with the eastern region taking the lead in development, the central region quickly rising, and the western region showing broad development prospects. In addition, the geographical proximity effect plays an important role in shaping the spatial dynamics of urban-rural integration development. As for the main influencing factors, broadband internet access, per capita disposable income, education expenditure, unemployment insurance coverage, and green coverage rate have become the core drivers of urban-rural integration development. Based on these findings, this paper suggests that the government should formulate differentiated development strategies based on different development stages, strengthen coordination and cooperation between regions, and adopt systematic development strategies to effectively address various challenges, thereby accelerating the process of urban-rural integration development.

Keywords: urban–rural integration; entropy weighting method; coupling coordination degree model; geographic detector model

1. Introduction

The relationship between urban and rural areas, as a fundamental relationship in human social development, has always been a significant theoretical and practical issue concerning the national economy and people's livelihoods(1). In the historical development of China, the relationship between urban and rural areas has undergone several transformations, which can be roughly summarized into three stages: urban-rural chaos, urban-rural opposition, and urban-rural integration(2). In the early stages of primitive society, due to the lack of technological means, the production methods were primitive and simple, highly dependent on nature's gifts. As a result, the boundary between cities and rural areas was not clearly defined, and there was no distinct urban-rural difference(3). However, with the rise and development of capitalism, cities quickly became the core of development, attracting a large influx of rural populations. Farmers gradually became wage workers within the capitalist production system, and a clear divide emerged between urban and rural areas in economic, social, and cultural aspects(4). Fortunately, continuous technological advancements brought new development opportunities to rural areas. The improvement in agricultural productivity freed up a large labor force in rural areas, which was transferred to more value-added industries, such as agricultural product processing and rural tourism,

bringing higher income levels to farmers(5). Meanwhile, the optimization and upgrading of the industrial structure not only enhanced the competitiveness of traditional agriculture but also gave birth to emerging sectors like modern agriculture and ecological agriculture, promoting the diversification of rural economies(6). Against this backdrop, urban-rural integration development gradually gained attention and became an important strategic direction for national development. In 2019, the Central Committee of the Communist Party of China and the State Council issued the "Opinions on Establishing and Improving the System and Policy Framework for Urban-Rural Integration Development," which clearly pointed out the need to accelerate the formation of a system mechanism for the smooth flow of urban and rural elements and the balanced allocation of public resources, to build a new type of urban-rural relationship that promotes mutual advancement between industry and agriculture, complements urban and rural areas, ensures coordinated development, and fosters common prosperity. Therefore, how to deeply understand the connotations of urban-rural integration development, accurately identify the practical challenges, and formulate effective policy measures to promote urban-rural integration development towards a more efficient, orderly, and sustainable path has become a major theoretical and practical issue that urgently requires in-depth exploration.

2. Literature Review

The concept of urban–rural integration derives from Thomas More’s seminal work, Utopia(1). In that work, More described an ideal society in which urban and rural regions were cohesively integrated, operating as a singular entity. In his concept, resource allocation, population distribution, and production activities were governed by need-based distribution, fostering societal resources’ equitable allocation and effective exploitation(7). This concept was as an intellectual precursor to subsequent urban–rural integration theories. At the end of the 19th century, Engels provided a more comprehensive theoretical framework in The Principles of Communism. He asserted that, to achieve urban–rural integration, antiquated labor divisions needed to be eliminated. He proposed that this could be achieved through cohesive production and education frameworks, facilitating adaptable employment transitions and an equal distribution of benefits, allowing all citizens to collaboratively generate and reap the rewards of shared prosperity. Engels also contended that such measures would promote the holistic development of human skills while enhancing urban–rural integration(2). A prominent early urban planner, Ebenezer Howard contributed to this discussion in Garden Cities of Tomorrow. By integrating urban–rural principles into his urban planning methodologies, Howard converted these concepts into implementable models, grounding the idea in actual applications(8). In sum, early urban–rural integration research, mainly influenced by Western utopian socialism, includes two fundamental perspectives: “urban-led rural development” and “rural-promoted urban development.” These perspectives provide a significant theoretical starting point for enhancing modern urban–rural integration strategies. On this basis, further theoretical doctrines have been developed, including the “Urban Bias Theory” “Rural Bias Theory” “Urban-Rural Dual Structure Theory” and “Urban-Rural Integrated Development Theory”.

As a multidimensional concept, urban–rural integration revolves around two entities: the “urban” and the “rural.” Urban areas, as unique geographical systems, are defined by aggregating non-agricultural enterprises and residents, whereas rural areas include all regions outside the urban limits(9). From a geographical standpoint, no distinct territorial boundary separates urban and rural regions. Their distinctions are predominantly evident in their functions rather than in their geographical arrangement. Cities deliver nonagricultural products, services, and related employment possibilities, whereas rural areas primarily focus on agricultural production, supplying agricultural products and services to society(10). This functional mismatch results in problems such as an obstructed flow of resources, inequitable resource distribution(11), delayed infrastructure development(12), fragmented industrial frameworks, and disproportionate public services(13), all of which substantially impede urban–rural integration.

To address these challenges and promote urban-rural integrated development, scholars have conducted in-depth research on urban-rural development from multiple dimensions, including agricultural development(14), healthcare services(15), ecological environment(16), human capital(17), and value concepts(18) between urban and rural areas. In terms of research methods, scholars have widely adopted various analytical techniques, such as factor analysis(19), principal component analysis(20) and intuitive fuzzy comprehensive evaluation(21), in an effort to scientifically measure the level of urban-rural integrated development. In terms of research scope, existing studies mainly focus on large-scale geographic spaces, such as urban agglomerations(22), metropolitan regions(23), and provinces or cities(24), particularly in economically developed regions like the Yellow River Basin, Dalian, Tianjin, and others, which are the primary hotspots of research. These studies provide a deep analysis of the urban-rural integration development models in these areas. In terms of research data, current studies primarily rely on official authoritative data sources such as the National Bureau of Statistics website and local statistical yearbooks. However, there is a lack of detailed data collection and analysis at the micro level, such as for villages or rural households. This may overlook the micro information within each evaluation unit, leading to biased results. Regarding research conclusions, scholars have proposed strategies such as promoting coordinated industrial development to foster mutual complementarity and win-win outcomes between urban and rural economies(25); striving for the equalization of public services to ensure that urban and rural residents enjoy equal social welfare(26); and emphasizing the strengthening of ecological environmental protection to ensure the sustainability of urban and rural development(27). These strategies together provide valuable references for promoting urban-rural integrated development.

In summary, research on urban–rural integration primarily focuses on theoretical foundations, core concepts, practical issues, and policy recommendations. These studies often rely on normative analytical frameworks, and the conclusions drawn are of considerable reference value. However, gaps remain in our understanding of urban–rural integration. First, some studies are limited to individual regions, lacking a comprehensive national-level study. Second, the research content is concentrated on the traditional dimensions of economy, society, and environment, neglecting the important aspects of infrastructure and social security and resulting in incomplete strategy recommendations. Therefore, we have taken the 31 provinces, autonomous regions, and municipalities

directly under the central government as the scope of our study and constructed a five-dimensional analytical framework that includes economics, society, ecology, infrastructure, and public services. Our aim is to provide a comprehensive measurement and systematic analysis of Chinese urban–rural integration over the past seven years. Figure 1 illustrates the specific steps of our research.

[Insert Figure 1]

Figure 1. Research structure.

3. Methods

3.1. Study Area

This study focuses on the 31 provinces, autonomous regions, and municipalities directly under the central government of China, specifically covering Beijing, Tianjin, Hebei, Shanxi, Inner Mongolia Autonomous Region, Liaoning, Jilin, Heilongjiang, Shanghai, Jiangsu, Zhejiang, Anhui, Fujian, Jiangxi, Shandong, Henan, Hubei, Hunan, Guangdong, Guangxi Zhuang Autonomous Region, Hainan, Chongqing, Sichuan, Guizhou, Yunnan, Tibet Autonomous Region, Shaanxi, Gansu, Qinghai, Ningxia Hui Autonomous Region, and Xinjiang Uygur Autonomous Region. It should be noted that, due to the unavailability of relevant data for Hong Kong Special Administrative Region, Macau Special Administrative Region, and Taiwan Province, these regions are not included in this study to ensure the scientific rigor and data accuracy of the research. The specific details are shown in Figure 2.

[Insert Figure 2]

Figure 2. Research area overview map.

3.2. Data Sources

The data used in this study primarily comes from official sources such as the National Bureau of Statistics, statistical data published by the Ministry of Industry and Information Technology, the China Statistical Yearbook, the China Urban and Rural Construction Statistical Yearbook, as well as statistical yearbooks from various provinces, cities, and autonomous regions. Additionally, the vector files of administrative boundaries were obtained from the Resources and Environmental Science and Data Center of the Chinese Academy of Sciences (website: https://www.resdc.cn/).

It is worth noting that during the data processing, a small amount of missing data was inevitably encountered. To address this issue, linear interpolation was employed to reasonably fill the gaps, ensuring data completeness and the continuity of the analysis. Compared to other methods, linear interpolation offers significant advantages when dealing with small amounts of missing data and

when the data generally exhibits a linear trend. It provides more precise approximations and effectively preserves the original characteristics of the data. Furthermore, due to its relatively simple calculation, linear interpolation avoids the additional errors that may arise from increased model complexity, thus enhancing both the efficiency of data processing and the accuracy of the analysis.

3.3. Indicator Descriptions

Urban–rural integration comprises a complex system with many influential factors such as infrastructure, economic development, public services, social security, and ecological environments(28). The movement and interaction among these factors within and across urban and rural areas constitute the fundamental mechanisms propelling the coordinated advancement of urban–rural integration.

Infrastructure constitutes the material foundation of urban–rural integration, supporting advancement and interconnectivity across geographies. Advanced physical infrastructure increases transit networks between urban and rural areas, facilitates information interchange, and improves the overall quality of life in both settings(29). To assess infrastructure development across geographical areas, we focused on several comparative coefficients. We used the comparative coefficient of urban-to-rural road length as the crucial indicator of the differences in transportation network configuration and road construction to assess the extent of physical infrastructure development(30). We used the comparative coefficient of broadband Internet access to reflect inequalities in information network infrastructure, representing a vital metric for urban–rural informatization(31); and we used the comparative coefficient of water supply coverage between urban and rural areas to reflect disparities in water infrastructure, which directly affect living conditions in these locations(32).

Economic development is a growth catalyst for urban–rural integration. Efficient economic growth enhances per capita income, improves industrial structures, and raises consumption levels in both urban and rural areas(33). To assess economic development across the provinces, we focused on three comparative coefficients. We used the comparative coefficient of per capita disposable income to demonstrate income differences and as a metric to assess the (dis)equilibrium of urban–rural economic development(34). We used the urban–rural dual comparison coefficient to assess the ratio of added value and employment in the primary sector to the secondary and tertiary industries to underscore disparities in industrial structures and facilitate the analysis of economic factors(35); and we used the comparative coefficient of total retail sales of consumer products to identify consumption differences between the urban and rural populations and provide insight into the integration of urban and rural consumer markets1.

Public services are essential for urban–rural integration. Public services need to be equitable, meet the education requirements of both urban and rural populations, elevate cultural standards, and improve transportation and communication networks(36). To assess public services across urban and rural areas, we again focused on three comparative coefficients. We used the comparative coefficient of per capita education expenditure between urban and rural populations to identify discrepancies in access to educational

resources and as a crucial statistic for assessing equity in urban–rural schooling(37). We used the comparative coefficient of per capita cultural and recreational expenditure to demonstrate disparities in cultural consumption levels and to provide insight into the integration of urban and rural cultural development(38); and we used the comparative coefficient of per capita transportation and communication expenditure to highlight connectivity and information-sharing inequalities and to assess the interaction between urban and rural areas(39).

Social security serves as a support system for both urban and rural populations, and thus is important for their integration. An effective social security system can mitigate issues associated with age-related care, health hazards, and unemployment, providing an equitable basis for urban–rural integration(40). To assess social security system development in the provinces, we focused again on three comparative coefficients. We used the comparative coefficient of pension insurance coverage to assess differences in older adult life security, functioning as a significant indicator of equity in urban–rural social welfare(37). We used the comparative coefficient of medical insurance coverage to capture disparities in health risks and assess access to medical services to evaluate the equity in urban–rural healthcare systems(41); and we used the comparative coefficient of unemployment insurance coverage to reflect differences in risk mitigation and reemployment assistance to assess employment security equity across urban and rural regions(42).

Ecological environment is fundamental for sustained urban–rural integration. An exemplary ecological environment improves citizens' quality of life and fosters green, sustainable development(43). To assess the ecological environments across the rural and urban areas, we focused on three comparative coefficients. We used the comparative coefficient of greening coverage to identify differences in green infrastructure, an essential metric of ecological integration(30). We used the comparative coefficient of sewage treatment rates to reflect environmental protection capabilities, offering insight into the degree of ecological preservation in urban and rural regions(44); and we used the comparative coefficient of household waste treatment rates to highlight the inequalities in domestic waste management and as a crucial tool for evaluating environmental governance(45). The details of these indicators are listed in Table 1.

Table 1. Evaluation indicator framework measuring urban–rural integration in China.

**Primary Indicator**	**Secondary Indicator**	**Indicator Name**	**Indicator Description**	**Attribute**	**Weight**
	Infrastructure	Urban–rural road length comparison coefficient	Urban road length / Rural road length / %	—	10.67%

		Urban–rural broadband Internet access comparison coefficient	Urban broadband Internet access users / Rural broadband Internet access users / %	−	3.47%
		Urban–rural water supply coverage comparison coefficient	Urban water supply coverage / Rural water supply coverage / %	−	4.98%
		Urban–rural per capita disposable income comparison coefficient	Per capita disposable income of urban residents / Per capita disposable income of rural people / %	−	10.22%
	Economic Development	Urban–rural dual comparison coefficient	(Value generated by primary industry / Number of primary industry workers) / (Value added by secondary and tertiary industries / Number of secondary and tertiary industry workers) / %	−	6.10%
Urban–Rural Integration		Urban–rural total retail sales of consumer goods comparison coefficient	Total retail sales of consumer goods in urban areas/Total retail sales of consumer goods in rural areas/ %	−	6.65%
		Urban–rural per capita education expenditure comparison coefficient	Per capita education expenditure of urban residents / Per capita education expenditure of rural people / %	−	3.36%
	Public Services	Urban–rural per capita cultural and recreational expenditure comparison coefficient	Per capita cultural and recreational spending of urban residents / Per capita cultural and recreational expenditure of rural people /%	−	10.26%
		Urban–rural per capita transportation and communication expenditure comparison coefficient	Per capita transportation and communication expenditure of urban people / per capita transportation and communication expenditure of rural residents/ %	−	5.45%
	Social Security	Urban–rural pension insurance coverage comparison coefficient	Coverage of basic pension insurance for urban and rural residents / Permanent population / %	+	4.66%

	Urban–rural medical insurance coverage comparison coefficient	Primary medical insurance coverage for urban and rural residents / Permanent population / %	+	6.86%
	Urban–rural unemployment insurance coverage comparison coefficient	Unemployment insurance coverage for urban and rural residents / Permanent population /%	+	4.33%
Ecological Environment	Urban–rural greening coverage comparison coefficient	Urban greening coverage ratio / Rural greening coverage ratio / %	—	8.70%
	Urban–rural sewage treatment rate comparison coefficient	Urban sewage treatment percentage / Rural sewage treatment percentage / %	—	8.20%
	Urban–rural household waste treatment rate comparison coefficient	Urban home waste management rate / Rural household waste management rate / %	—	6.08%

3.4. Modeling

3.4.1. Entropy Weighting

The entropy weighting method is an objective weighting technique commonly employed in complex assessments(46). This method computes the entropy of the indicators to evaluate the quantity of information they provide, establishing their corresponding weights in the assessment process. Indicators exhibiting elevated entropy signify increased system disorder and convey less information, resulting in reduced weights, whereas indications with lower entropy denote enhanced system order and better information, resulting in higher weights. Compared with conventional expert scoring techniques, the entropy method eliminates the subjectivity associated with manual scoring, thereby enhancing the reliability and objectivity of the data analysis. The precise steps are as follows:

Determine the ratio of indicators P_{ij}:

$$P_{ij} = \frac{X'_{ij}}{\sum_{i=1}^{n} X'_{ij}} \quad (1)$$

Compute the entropy of the indicators E_j:

$$E_j = -K \sum_{i=1}^{n} P_{ij} ln P_{ij} \quad (2)$$

Compute the information utility value D_j:

$$D_j = 1 - E_j \quad (3)$$

Determine the weights W_j:

$$W_j = 1 - \frac{E_j}{\sum_{j=1}^{m} D_j} \tag{4}$$

where i denotes the ith year; j represents the jth indicator; k=1/ln^n, where n signifies the number of years; $0 \le E_j \le 1$; if $P_{ij}=0$, then assign $P_{ij}\ln P_{ij}=0$.

3.4.2. Coupling Coordination Degree

The coupling coordination degree model is a quantitative analytical instrument used to evaluate the extent of mutual effects and interactions among two or more systems. The model includes a coupling coordination function that amalgamates the individual developmental level of each system and their interconnections, thus ascertaining the overall state of coupling and coordination among the systems(47). In contrast to other analytical methods, the coupling coordination degree model measures the intensity of interconnections across systems and reveals the coordination dynamics among the different elements in each system. The precise equations are as follows:

$$C = 5 \times \left\{ \frac{U_1 \times U_2 \times U_3 \times U_4 \times U_5}{(U_1 + U_2 + U_3 + U_4 U_5)^2} \right\}^{\frac{1}{5}} \tag{5}$$

$$T = \beta_1 U_1 + \beta_2 U_2 + \beta_3 U_3 + \beta_4 U_4 + \beta_5 U_5 \tag{6}$$

$$D = \sqrt{C \times T} \tag{7}$$

where C denotes the coupling degree; T is the comprehensive development level; and D is the coupling coordination degree. U_1, U_2, U_3, U_4 and U_5 denote the composite index scores for infrastructure, economic development, public services, social security, and the ecological environments, respectively. $\beta_1, \beta_2, \beta_3, \beta_4$ and β_5 are the coefficients of interest. We assume that the five sub-systems hold equal significance; hence, we allocate a value of 1/5 for each. By establishing classification criteria in coordination degree research, the coordination degree can be categorized into 10 classifications, as shown in Table 2.

Table 2. Criteria for classifying the degree of coupling coordination.

Coupling Coordination Degree D Value	Coordination Type	Coupling Coordination Degree D Value	Coordination Type
0.0~0.1	Extreme Discrepancy	0.5~0.6	Slight Coordination
0.1~0.2	Severe Discrepancy	0.6~0.7	Mild Coordination
0.2~0.3	Moderate Discrepancy	0.7~0.8	Moderate Coordination

0.3~0.4	Mild Discrepancy	0.8~0.9	High Coordination
0.4~0.5	Slight Discrepancy	0.9~1.0	Extreme Coordination

3.4.3. Geographic Detector Model

The geographic detector is a statistical instrument that identifies the principal elements affecting geographic phenomena. Based on spatial heterogeneity, it measures the intensity of spatial correlations between variables and geographic phenomena, thereby assessing the impact of each variable on the spatial patterns(48). In contrast to conventional techniques, the geographic detector model evaluates the individual impact of each component and reveals the interactions among many factors, thereby providing a more thorough understanding of the underlying mechanisms. The precise equation is as follows:

$$q = 1 - \frac{1}{N\sigma^2}\sum_{h=1}^{L} N_h \sigma_h^2 \quad (8)$$

where q denotes the value of the detector factor; h=1; L denotes the stratification of variable X; N and N_h signify the sample sizes of the study region and detection area, respectively; and σ^2 and σ_h^2 indicate the variance of variable Y in the study area and detection area, respectively. The q value ranges from 0 to 1, where a better q value signifies a more robust explanatory influence of factor X on variable Y.

4. Results

Our analysis examined comprehensive data to assess the progress of urban–rural integration levels in various regions of China, carefully considering the coordination degree values from 2015 to 2022. Our results offer insight into the efficacy of regional initiatives aimed at promoting urban–rural integration and establishes a solid basis for guiding future policy decisions. We summarize the integration degree by year in Table 3.

Table 3. Index of urban–rural integration development across China regions from 2015 to 2020.

Region	2015	2016	2017	2018	2019	2020	2021	2022
Beijing	0.343	0.414	0.528	0.893	0.825	0.439	0.49	0.429
Tianjin	0.138	0.336	0.588	0.824	0.847	0.872	0.935	0.941
Hebei	0.348	0.341	0.427	0.438	0.416	0.627	0.367	0.525
Shanxi	0.518	0.631	0.376	0.4	0.671	0.48	0.474	0.408
Inner Mongolia	0.441	0.396	0.404	0.485	0.588	0.391	0.419	0.265
Liaoning	0.417	0.346	0.34	0.553	0.796	0.467	0.436	0.404
Jilin	0.336	0.878	0.363	0.478	0.639	0.495	0.437	0.225
Heilongjiang	0.599	0.752	0.684	0.604	0.488	0.601	0.466	0.25

Shanghai	0.239	0.631	0.656	0.557	0.696	0.759	0.698	0.397
Jiangsu	0.278	0.34	0.342	0.491	0.659	0.672	0.625	0.397
Zhejiang	0.535	0.73	0.735	0.561	0.628	0.458	0.617	0.221
Anhui	0.517	0.523	0.509	0.409	0.555	0.286	0.643	0.602
Fujian	0.388	0.584	0.745	0.507	0.56	0.327	0.361	0.422
Jiangxi	0.455	0.38	0.405	0.671	0.566	0.324	0.539	0.368
Shandong	0.379	0.249	0.418	0.444	0.453	0.551	0.779	0.508
Henan	0.305	0.264	0.589	0.56	0.49	0.469	0.689	0.432
Hubei	0.415	0.553	0.544	0.374	0.35	0.34	0.42	0.422
Hunan	0.48	0.441	0.754	0.451	0.341	0.556	0.555	0.383
Guangdong	0.402	0.273	0.523	0.614	0.716	0.549	0.489	0.375
Guangxi	0.543	0.611	0.584	0.437	0.563	0.463	0.299	0.159
Hainan	0.514	0.686	0.512	0.809	0.832	0.419	0.409	0.275
Chongqing	0.841	0.637	0.438	0.423	0.402	0.337	0.294	0.158
Sichuan	0.215	0.342	0.413	0.366	0.356	0.629	0.715	0.628
Guizhou	0.659	0.542	0.366	0.564	0.631	0.397	0.459	0.547
Yunnan	0.403	0.458	0.521	0.415	0.39	0.365	0.362	0.252
Tibet	0.445	0.357	0.257	0.417	0.401	0.325	0.339	0.256
Shaanxi	0.43	0.386	0.32	0.59	0.496	0.483	0.72	0.392
Gansu	0.355	0.411	0.62	0.477	0.753	0.622	0.55	0.386
Qinghai	0.633	0.575	0.936	0.49	0.614	0.464	0.298	0.213
Ningxia	0.524	0.714	0.603	0.478	0.325	0.418	0.355	0.227
Xinjiang	0.491	0.56	0.702	0.642	0.475	0.316	0.337	0.213

4.1. Temporal Evolution of Urban–Rural Integration Development in China

We found that the trajectory of urban–rural integration in China showed an upward tendency, although with unequal growth rates, typified by a pattern with phases of rapid growth, slow development, and a fluctuating rise. During the rapid expansion phase (2015–2018), the urban–rural integration coupling coordination degree increased markedly from 0.2931 to 0.5510, reflecting an average growth rate of 22.64%. The most significant advancement occurred from 0.4092 to 0.5510, indicating an impressive increase of 34.65%, the highest singular growth in this period. This signified a pivotal phase of rapid urban–rural convergence. In contrast, the gradual development period (2018–2019) experienced a minimal increase in the coupling coordination degree, from 0.5510 to 0.5520, with an average growth rate of merely 0.18%. This phase showed minimal growth throughout all intervals, indicating a

developmental bottleneck in which integration slowed markedly. The fluctuating phase (2019–2022) reflected new acceleration in urban–rural integration, with the coupling coordination degree increasing from 0.5520 to 0.8141, at an average growth rate of 13.99%. This phase was also volatile. The initial interval, ranging from 0.5520 to 0.6746, saw substantial growth of 22.21%, followed by a decrease from 0.6746 to 0.7227, where the growth fell to 7.13%, suggesting growth fatigue. However, the concluding interval, ranging from 0.7227 to 0.8141, showed a reacceleration in growth to 12.65%. Overall, this phase represented a general upward trend despite volatility and inconsistent progress. Figure 3 illustrates these trends.

[Insert Figure 3]

Figure 3. The temporal changes in urban–rural integration development in China (2015–2022).

4.2. Spatial Evolution of Urban–Rural Integration Development in China

We found several spatial differences in China's urban–rural integration development, resulting in a varied pattern in which the eastern coastal regions dominated rural–urban integration, the central regions showed swift advancement, and the western regions demonstrate considerable potential. Our analysis reflected an important geographical proximity effect, wherein adjacent regions were able to exert reciprocal influence and support for each other, encouraging a tendency for regional interconnectedness development.

In 2015, the eastern coastal areas spearheaded the urban–rural integration growth owing to their strategic geographic position, solid economic base, and openness. Tianjin (0.625), Guangdong (0.559), and Zhejiang (0.455) exhibited urban–rural integration indices highly exceeding the national average, establishing them as national benchmarks. By 2019, the eastern regions' swift development had generated a radiative effect, catalyzing progress in the central region. Based on their vast natural resources and labor force, these regions saw significant advancement, with Anhui Province (0.930) attaining the highest integration score. Hubei (0.722) and Hunan (0.708) were close behind, emerging as critical regions for urban–rural integration development. The 2022 national policies supporting western regions are expected to encourage further development. Yunnan (0.941), the Guangxi Zhuang Autonomous Region (0.935), and the Tibet Autonomous Region (0.933) had elevated integration indices, highlighting these western areas' significant potential, signifying a new stage in Chinese urban–rural interactions.

Geographical proximity's influence was apparent across many provinces. Zhejiang, a frontrunner in urban–rural integration with a development index of 0.662, attained economic prosperity and social advancement while impacting nearby Jiangsu (0.648) and Anhui (0.758), enhancing their rural–urban integration. This effect transcends the eastern regions. Sichuan Province, a pivotal western-area contributor, was a robust driving force with an integration level of 0.678, promoting advancement in adjacent Yunnan (0.677) and Guizhou (0.722). Thus, interrelated urban–rural integration development dynamics appear nationwide (Figure 4-6).

[Insert Figure 4]

Figure 4. The spatial distribution of urban–rural integration development in 2015.

[Insert Figure 5]

Figure 5. The spatial distribution of urban–rural integration development in 2019.

[Insert Figure 6]

Figure 6. The spatial distribution of urban–rural integration development in 2022.

4.3. Primary Driving Factors of Urban–Rural Integration Development in China

We examined several main factors (infrastructure, economic development, public services, social security, and the ecological environments) that are expected to have a significant effect on urban–rural integration in China. We found that among these, the most significant drivers were broadband Internet availability, per capita disposable income, education spending, unemployment insurance coverage, and green ecological coverage. Within the infrastructure category (X_1–X_3), the urban–rural broadband Internet access comparison coefficient (X_2) had the highest q value of 0.6498. In economic development (X_4–X_6), the urban–rural per capita disposable income comparison coefficient (X_4) had the highest q value of 0.5258. In public services (X_7–X_9), the coefficient for the urban–rural per capita education spending (X_7) was notable, with a q value of 0.4360. In social security (X_{10}–X_{12}), the comparative coefficient for urban–rural unemployment insurance coverage (X_{12}) had the highest q value of 0.5198. Finally, in the ecological environment (X_{13}–X_{15}), the urban–rural green coverage comparison coefficient (X_{13}) had the highest q value at 0.4213. These findings highlight the unique contributions of each factor in urban–rural integration. These results are shown in Figure 7.

[Insert Figure 7]

Figure 7. The main driving factors of urban–rural integration in China (2015–2022).

5. Discussion

Urban–rural integration in China has shown overall improvement but with inconsistent progress, marked by periods of rapid growth, slow development, and fluctuating increases. This pattern can be attributed to three main factors. First, policy support is crucial for driving urban–rural integration. This viewpoint aligns with the perspective in Harris JK 's research, which highlights the important role of public policies in promoting the development of both urban and rural areas(49). Therefore, the Chinese government has issued key policies, such as "Several Opinions on Increasing Reform and Innovation Efforts to Accelerate Agricultural Modernization" and "Several Opinions on Deepening Agricultural Supply-Side Structural Reform," which provide financial investment and resource allocation to boost rural development. These policies have enhanced rural economic growth, facilitated the flow of production factors, and expanded urban and rural markets, laying a solid foundation for integration. Second, the COVID-19 pandemic, which began in 2019, created significant barriers to urban–rural integration. This situation aligns with the analysis in Jiang

X 's research on the impact of external shocks on urban-rural development(50). Strict traffic restrictions and lockdowns disrupted the flow of goods, especially agricultural products, leading to unsold surpluses, reduced farmer incomes, and increased urban–rural economic disparities. This setback hindered the progress of integration. Third, structural changes in the economy have also impacted urban–rural integration. This viewpoint echoes the research of Cheng X(51). As China shifts toward high-quality development, traditional industries face transformation challenges, while emerging industries are not yet large enough to offset their decline. This transition has intensified resource reallocation and competition in urban areas, weakened rural agricultural markets, and exacerbated urban–rural inequalities, complicating the integration process.

Urban–rural integration in China exhibits significant regional variation. The eastern coastal areas have made rapid progress, benefiting from advantageous geography, large transportation networks, and active participation in international trade. This is demonstrated in Zhao Y's research(51). The central region has also seen swift development, leveraging spillover effects from the eastern areas to advance its industries. In contrast, the western region, with its inland terrain and challenging ecological conditions, has less-developed infrastructure. However, recent government investments in infrastructure and poverty reduction have created new growth opportunities, enhancing logistical efficiency and improving living conditions, thus showing potential for integration, which is consistent with the conclusions of existing research(1). Geographical proximity plays a significant role in urban–rural integration. Adjacent regions with similar infrastructure promote efficient resource movement and distribution. This point is demonstrated in Xia S's research(52). High-performing regions support underdeveloped areas through spillover effects, fostering coordinated development.

Key determinants of urban–rural integration include broadband Internet accessibility, per capita disposable income, educational expenditure, unemployment insurance coverage, and green coverage. Broadband connectivity is essential for information flow, knowledge acquisition, and cultural interaction. However, rural areas often lag behind urban centers due to weak economic foundations and inadequate infrastructure(53). Bridging this digital divide is crucial for equitable development. Per capita disposable income is a vital metric for comparing regional development and reflects consumption capability and living standards. Rural incomes are often lower due to limited industries and outdated production methods, restricting consumption and economic growth(54). Addressing this disparity is key to enhancing rural vitality and integration. Educational investment is crucial for improving human capital quality. Disproportionate allocation of education resources is a primary cause of urban–rural disparities(55). Increased investment in rural education could improve educational achievements and employment opportunities, while addressing the resource deficit is essential for overall human capital development. Unemployment insurance coverage is a measure of social security. Rural areas typically have lower coverage due to economic weakness and lack of institutional capacity, making inhabitants more vulnerable to unemployment risks(56). Enhancing coverage in rural regions is essential for social stability and integration. Green coverage is a key indicator of ecological quality. Better green coverage in urban areas restricts rural populations' access to ecological benefits and hampers environmental improvement(57). Reducing this disparity is crucial for improving rural living conditions and facilitating integration.

Based on the above analysis, we propose the following policy recommendations to promote the improvement of the level of urban-rural integration development in China.

(1) Driven by policy initiatives, economic development, and ongoing social demands, urban–rural integration in China has shown an overall upward trend. However, its development is uneven, characterized by phases of rapid growth, gradual progress, and fluctuating advancement, highlighting the need for flexible and targeted policy measures. During a rapid growth phase, the government should emphasize enhanced investment and optimized resource allocation to provide a robust foundation for integration. During a gradual development phase, the focus should be on resolving fundamental structural issues, including transforming rural industries and improving employment prospects and income for farmers. Strategic policy directions and assistance are essential for rejuvenating rural economies and promoting equitable development. During a volatile ascent period, it is crucial to closely observe market dynamics and potential dangers and enhance macroeconomic regulation and policy coordination to ensure consistent advancement and alleviate disturbances in the integration process.

(2) The government should implement diversified development policies to reduce regional disparities in urban–rural integration. In the eastern coastal areas, priority should be given to maintaining their leading position and advancing urban–rural integration to a higher level. In contrast, strengthening infrastructure construction and improving public service systems are essential for the inland regions of central and western China. Additionally, promoting cross-regional collaboration and communication frameworks is crucial. Eliminating regional barriers, facilitating unrestricted movement, and effectively allocating resources, technology, and talent will help create synergetic advantages and accelerate the development of a cooperative and progressive regional development model.

(3) Given that urban–rural integration in China is influenced by multiple variables, the government should implement a systematic and comprehensive development strategy to address these issues. Investment in infrastructure should prioritize rural areas and upgrade information infrastructure, such as broadband connectivity, to facilitate improved information flow and resource sharing across urban and rural regions. The government should also advocate for the sustainable utilization of local rural resources by endorsing specialty agriculture and rural tourism, thereby enhancing farmers' per capita disposable income and reinforcing the material base for integration. Investment in education, healthcare, and other vital sectors in rural areas should be increased to strengthen service systems, thereby enhancing the quality of life and well-being of urban and rural populations. Social security should emphasize improvement in unemployment insurance and its associated systems to provide extensive and resilient social protections for everyone. The government should also prioritize environmental conservation and governance, pushing efforts such as afforestation and urban greening projects, to foster healthier and more habitable environments for both urban and rural inhabitants. By implementing this comprehensive suite of targeted policies, the government can more effectively promote urban–rural integration, address inequities, and facilitate sustainable and balanced growth throughout China.

6. Conclusions

Based on the panel data of 31 provinces, autonomous regions, and municipalities directly under the central government in China from 2015 to 2022, we used the entropy method to comprehensively measure the comprehensive index of urban-rural integration development in each region. Subsequently, we applied the coupling coordination degree model to analyze the coordination level of urban-rural integration development in each region. On this basis, we further analyzed the spatiotemporal evolution characteristics of urban-rural integration development in China in detail from both temporal and spatial perspectives. Finally, using the geographical detector model, we empirically analyzed the main driving factors affecting urban-rural integration development in China. The main conclusions are as follows:

(1) From a temporal perspective, the overall trend of urban-rural integration development in China shows an upward trajectory, but the growth rate is uneven, exhibiting a wave-like pattern of "rapid growth—slow development—fluctuating increase."

(2) From a spatial perspective, the spatial disparity in urban-rural integration development in China is significant, forming a diverse spatial development pattern with the eastern coastal regions leading in development, the central region rapidly rising, and the western region having huge potential. In this process, the geographical proximity effect is notable, with neighboring regions being interdependent and mutually promoting each other in the urban-rural integration development process, creating a regional linkage development trend.

(3) From the perspective of driving factors, the urban-rural integration development in China is influenced by infrastructure, economic development, public services, social security, and the ecological environment. Among these, internet broadband access, per capita disposable income, education expenditure, unemployment insurance coverage, and greening coverage play the main driving roles.

The potential innovation of this research lies in abandoning the traditional paradigm of viewing urban and rural areas as two separate units and separately addressing their respective development issues. Instead, it employs a systems theory approach, viewing them as a closely connected and mutually interactive organic whole. By deeply investigating the development level, core characteristics, and influencing factors of urban-rural integration, this research enhances the understanding of the internal mechanisms of urban-rural integration and provides a solid empirical foundation for formulating strategies for urban-rural integration development. On this basis, the study further expands the traditional three-dimensional analytical framework of economy, society, and ecology, innovatively incorporating two additional dimensions: infrastructure and public services, thus constructing a five-dimensional analytical framework encompassing economy, society, ecology, infrastructure, and public services. This upgrade not only significantly enhances the comprehensiveness of urban-rural integration development level assessment but also provides policymakers with a more detailed reference framework, helping to implement more scientific, rational, and efficient policy measures.

However, this study also encountered several challenges during its progression, which should be addressed in future research. On one hand, although this study strives to comprehensively reveal the current state of urban-rural integration development, it is still difficult to cover all its aspects, such as population mobility, land use, and cultural integration, which to some extent limits the depth and breadth of the research. On the other hand, this study mainly relies on statistical yearbook data, and the inherent update

cycle of these data results in a significant time lag. Currently, the data is only updated until 2022, which undoubtedly limits the in-depth exploration of the latest trends in urban-rural integration development, thus affecting the research's foresight and timeliness. In response to these challenges, future research can improve in two aspects. On one hand, the urban-rural integration development evaluation index system should be further refined and optimized, incorporating more areas closely related to urban-rural integration development to more comprehensively and accurately reflect the real situation of urban-rural integration development. On the other hand, efforts should be made to actively collect and utilize the latest statistical data to keep up with the latest trends in urban-rural integration development, thereby enhancing the timeliness and foresight of the research.

References:

1.Wei L, Zhao X, Lu J. Measuring the Level of Urban-Rural Integration Development and Analyzing the Spatial Pattern Based on the New Development Concept: Evidence from Cities in the Yellow River Basin. Int J Env Res Pub He. 2022;20(1). http://doi.org/10.3390/ijerph20010015

2.Sun X, Chen J, Xie S. Becoming Urban Citizens: A Three-Phase Perspective on the Social Integration of Rural-Urban Migrants in China. Int J Env Res Pub He. 2022;19(10). http://doi.org/10.3390/ijerph19105946

3.Zhang S, Chen W, Li Q, Li M. Evaluation and development strategy of urban-rural integration under ecological protection in the Yellow River Basin, China. Environ Sci Pollut R. 2023;30(40):92674-91. http://doi.org/10.1007/s11356-023-28950-7

4.Miao J, Wu X. Urbanization, socioeconomic status and health disparity in China. Health Place. 2016;42:87-95. http://doi.org/10.1016/j.healthplace.2016.09.008

5.Yao S, Wu G. Research on the Efficiency of Green Agricultural Science and Technology Innovation Resource Allocation Based on a Three-Stage DEA Model-A Case Study of Anhui Province, China. Int J Env Res Pub He. 2022;19(20). http://doi.org/10.3390/ijerph192013683

6.Wei Q, Jian C. Impact of non-agricultural employment on industrial structural upgrading -Based on the household consumption perspective. Plos One. 2024;19(2):e0294333. http://doi.org/10.1371/journal.pone.0294333

7.Luo G, Yang Y, Wang L. Driving rural industry revitalization in the digital economy era: Exploring strategies and pathways in China. Plos One. 2023;18(9):e0292241. http://doi.org/10.1371/journal.pone.0292241

8.Sun Y, Ding G, Li M, Zhang M, Agyeman FO, Liu F. The spillover effect of green finance development on rural revitalization: an empirical analysis based on China's provincial panel data. Environ Sci Pollut R. 2023;30(20):58907-19. http://doi.org/10.1007/s11356-023-26655-5

9.Zhao Y, Li J, Liu K, Shang C. Impact of urban-rural development and its industrial elements on regional economic growth: An analysis based on provincial panel data in China. Heliyon. 2024;10(16):e36221. http://doi.org/10.1016/j.heliyon.2024.e36221

10.Cheng X, Xu D, Sun H, Zheng M, Li J. Rural Spatial Differentiation and Revitalization Approaches in China: A Case Study of Qingdao City. Int J Env Res Pub He. 2022;19(24). http://doi.org/10.3390/ijerph192416924

11.Shi C, Zhu X, Wu H, Li Z. Urbanization Impact on Regional Sustainable Development: Through the Lens of Urban-Rural Resilience. Int J Env Res Pub He. 2022;19(22). http://doi.org/10.3390/ijerph192215407

12.Qiao G, Wang L, Du P. Contradiction or harmony? Spatial and temporal relationships between new urbanization and rural revitalization in the Yellow River Basin from a coupling perspective. Plos One. 2023;18(7):e0288600. http://doi.org/10.1371/journal.pone.0288600

13.Pan M, Huang Y, Qin Y, Li X, Lang W. Problems and Strategies of Allocating Public Service Resources in Rural Areas in the Context of County Urbanization. Int J Env Res Pub He. 2022;19(21). http://doi.org/10.3390/ijerph192114596

14.Smyth SJ, Charlebois S. Agricultural chemical use and the rural-urban divide in Canada. Gm Crops Food. 2024;15(1):32-9. http://doi.org/10.1080/21645698.2024.2318876

15.Chi Z, Han H. Urban-Rural Differences: The Impact of Social Support on the Use of Multiple Healthcare Services for Older People. Front Public Health. 2022;10:851616. http://doi.org/10.3389/fpubh.2022.851616

16.Nazari M, Rakhshani T, Jeihooni AK, Abnoos AB, Asadollahi A. Environment Intensifies Imbalance and Fear of Falling: Comparing Urban and Rural Aging Communities. Curr Aging Sci. 2023;16(3):211-8. http://doi.org/10.2174/1874609816666230503094432

17.Miller MK, Dixon BL, Fendley K. The economic costs and benefits of adding medical manpower to rural and urban communities: a human capital perspective. J Rural Health. 1986;2(2):17-36. http://doi.org/10.1111/j.1748-0361.1986.tb00143.x

18.Yang F, Jiang Y, Paudel KP. Impact of Work Value Awareness on Self-Rated Physical Health of Rural-to-Urban Migrant Workers in China. Healthcare-Basel. 2021;9(5). http://doi.org/10.3390/healthcare9050505

19.Kim D, Peterson N, Lee JE. Caregiving Outcomes of Sub/Urban and Rural Caregivers: The Powerful Tools for Caregivers Program. Clin Gerontologist. 2024;47(5):897-908. http://doi.org/10.1080/07317115.2022.2090878

20.Rifat SAA, Liu W. One year into the pandemic: the impacts of social vulnerability on COVID-19 outcomes and urban-rural differences in the conterminous United States. Int J Environ Heal R. 2022;32(12):2601-19. http://doi.org/10.1080/09603123.2021.1979196

21.Li F, Yan J, Xiong X, Yan H, Tao T, Wang L. GIS-based fuzzy comprehensive evaluation of urban flooding risk with socioeconomic index system development. Environ Sci Pollut R. 2023;30(18):53635-47. http://doi.org/10.1007/s11356-023-25972-z

22.Wei L, Zhao X, Lu J. Measuring the Level of Urban-Rural Integration Development and Analyzing the Spatial Pattern Based on the New Development Concept: Evidence from Cities in the Yellow River Basin. Int J Env Res Pub He. 2022;20(1). http://doi.org/10.3390/ijerph20010015

23.Qiu Y, Lu W, Guo J, Sun C, Liu X. Examining the Urban and Rural Healthcare Progress in Big Cities of China: Analysis of Monitoring Data in Dalian from 2008 to 2017. Int J Env Res Pub He. 2020;17(4). http://doi.org/10.3390/ijerph17041148

24.Luo D, Du J, Wang P, Yang W. Urban-rural comparisons in health risk factor, health status and outcomes in Tianjin, China: A cross-sectional survey (2009-2013). Aust J Rural Health. 2019;27(6):535-41. http://doi.org/10.1111/ajr.12562

25.Dong Y, Guo B, He D, Liao X, Zhang Z, Wu X. Industrial Transformation and Urban Economic Efficiency Evolution: An Empirical Study of the Yangtze River Economic Belt. Int J Env Res Pub He. 2022;19(7). http://doi.org/10.3390/ijerph19074154

26.Mousseau DY, Mousseau M. Allocation of public health services across urban and rural regions and armed conflicts in Sub-Saharan Africa. J Public Health Pol. 2024;45(3):460-70. http://doi.org/10.1057/s41271-024-00472-7

27.Hendryx M, Fedorko E, Halverson J. Pollution sources and mortality rates across rural-urban areas in the United States. J Rural Health. 2010;26(4):383-91. http://doi.org/10.1111/j.1748-0361.2010.00305.x

28.Zhang S, Chen W, Li Q, Li M. Evaluation and development strategy of urban-rural integration under ecological protection in the Yellow River Basin, China. Environ Sci Pollut R. 2023;30(40):92674-91. http://doi.org/10.1007/s11356-023-28950-7

29.Hu Z, Song G, Hu Z, Zhang B, Lin T. How to promote the balanced development of urban and rural China? Evidences from reallocating idle rural residential land of Zhejiang province, China. Plos One. 2023;18(6):e0287820. http://doi.org/10.1371/journal.pone.0287820

30.Xu C, Qian C, Yang W, Li B, Kong L, Kong F. Spatiotemporal Pattern of Urban-Rural Integration Development and Its Driving Mechanism Analysis in Hangzhou Bay Urban Agglomeration. Int J Env Res Pub He. 2022;19(14). http://doi.org/10.3390/ijerph19148390

31.Deng X, Guo M, Liu Y. Digital economy development and the urban-rural income gap: Evidence from Chinese cities. Plos One. 2023;18(2):e0280225. http://doi.org/10.1371/journal.pone.0280225

32.Liu Y, Qiao J, Xiao J, Han D, Pan T. Evaluation of the Effectiveness of Rural Revitalization and an Improvement Path: A Typical Old Revolutionary Cultural Area as an Example. Int J Env Res Pub He. 2022;19(20). http://doi.org/10.3390/ijerph192013494

33.Wineman A, Alia DY, Anderson CL. Definitions of "rural" and "urban" and understandings of economic transformation: Evidence from Tanzania. J Rural Stud. 2020;79:254-68. http://doi.org/10.1016/j.jrurstud.2020.08.014

34.Liu Q, Gong D, Gong Y. Index system of rural human settlement in rural revitalization under the perspective of China. Sci Rep-Uk. 2022;12(1):10586. http://doi.org/10.1038/s41598-022-13334-7

35.Zhang R, Ma C, Wu D, Wu Y, Wang K. The Evaluation and Optimization Methods of Villages in China: In the Background of a Rural Revitalization Strategy. Comput Intel Neurosc. 2022;2022:7314446. http://doi.org/10.1155/2022/7314446

36.Wang Y, Zhu H, Aziz N, Liu Y. Does Social Capital Improve the Effectiveness of Public Service? An Insight from Rural-to-Urban Migrants in China. Soc Indic Res. 2023;165(2):431-52. http://doi.org/10.1007/s11205-022-03021-5

37.Li Z, Liu C, Chen X. Power of Digital Economy to Drive Urban-Rural Integration: Intrinsic Mechanism and Spatial Effect, from Perspective of Multidimensional Integration. Int J Env Res Pub He. 2022;19(23). http://doi.org/10.3390/ijerph192315459

38.Mynarikova L, Posta V. The Effect of Consumer Confidence and Subjective Well-being on Consumers' Spending Behavior. J Happiness Stud. 2023;24(2):429-53. http://doi.org/10.1007/s10902-022-00603-5

39.Liu W, Wang J. Democracy, information, and communication technology infrastructure and environmental quality. Environ Sci Pollut R. 2023;30(48):105259-74. http://doi.org/10.1007/s11356-023-29850-6

40.Hu Y, Wu Y, Zhou W, Li T, Li L. A three-stage DEA-based efficiency evaluation of social security expenditure in China. Plos One. 2020;15(2):e0226046. http://doi.org/10.1371/journal.pone.0226046

41.Huo J, Hu M, Li S. The impact of urban-rural medical insurance integration on medical impoverishment: evidence from China. Int J Equity Health. 2023;22(1):245. http://doi.org/10.1186/s12939-023-02063-6

42.Tefft N. Insights on unemployment, unemployment insurance, and mental health. J Health Econ. 2011;30(2):258-64. http://doi.org/10.1016/j.jhealeco.2011.01.006

43.Sherpa TO. Integration of urban ecosystem-based adaptation in Nepal: A policy landscape analysis. Plos One. 2024;19(1):e0297786. http://doi.org/10.1371/journal.pone.0297786

44.Cheng P, Jin Q. Performance evaluation of the emerging rural sewage treatment facilities in China. Environ Sci Pollut R. 2022;29(34):51623-34. http://doi.org/10.1007/s11356-022-19467-6

45.Han Z, Liu Y, Zhong M, Shi G, Li Q, Zeng D, et al. Influencing factors of domestic waste characteristics in rural areas of developing countries. Waste Manage. 2018;72:45-54. http://doi.org/10.1016/j.wasman.2017.11.039

46.Tan X, Yu H, An Y, Wang Z, Jiang L, Ren H. Spatial Differentiation and Influencing Factors of Poverty Alleviation Performance Under the Background of Sustainable Development: A Case Study of Contiguous Destitute Areas in Hunan Province, China. Chinese Geogr Sci. 2021;31(6):1029-44. http://doi.org/10.1007/s11769-021-1242-4

47.Shuang L, Ximing C, Jie S. Research on the degree of coupling of the Internet development level and agricultural--Ecological efficiency based on 2009-2018 data from 13 major grain-producing areas in China. Plos One. 2021;16(7):e0254078. http://doi.org/10.1371/journal.pone.0254078

48.Shan B, Zhang Q, Ren Q, Yu X, Chen Y. Spatial heterogeneity of urban-rural integration and its influencing factors in Shandong province of China. Sci Rep-Uk. 2022;12(1):14317. http://doi.org/10.1038/s41598-022-18424-0

49.Harris JK, Mueller NL. Policy activity and policy adoption in rural, suburban, and urban local health departments. J Public Health Man. 2013;19(2):E1-8. http://doi.org/10.1097/PHH.0b013e318252ee8c

50.Jiang X, Wei W, Wang S, Zhang T, Lu C. Effects of COVID-19 on Urban Population Flow in China. Int J Env Res Pub He. 2021;18(4). http://doi.org/10.3390/ijerph18041617

51.Cheng X, Xu D, Sun H, Zheng M, Li J. Rural Spatial Differentiation and Revitalization Approaches in China: A Case Study of Qingdao City. Int J Env Res Pub He. 2022;19(24). http://doi.org/10.3390/ijerph192416924

52.Xia S, Yang Y, Qian X, Xu X. Spatiotemporal Interaction and Socioeconomic Determinants of Rural Energy Poverty in China. Int J Env Res Pub He. 2022;19(17). http://doi.org/10.3390/ijerph191710851

53.Mahmood S, Ali G, Menhas R, Sabir M. Belt and road initiative as a catalyst of infrastructure development: Assessment of resident's perception and attitude towards China-Pakistan Economic Corridor. Plos One. 2022;17(7):e0271243. http://doi.org/10.1371/journal.pone.0271243

54.Deng X, Guo M, Liu Y. Digital economy development and the urban-rural income gap: Evidence from Chinese cities. Plos One. 2023;18(2):e0280225. http://doi.org/10.1371/journal.pone.0280225

55.Guo Y, Li X. Regional inequality in China's educational development: An urban-rural comparison. Heliyon. 2024;10(4):e26249. http://doi.org/10.1016/j.heliyon.2024.e26249

56.Zhao Y, Zhou Z, Fan X, Nawaz R, Zhao D, Xu T, et al. Comparison of inequity in health-related quality of life among unemployed and employed individuals in China. Bmc Public Health. 2021;21(1):52. http://doi.org/10.1186/s12889-020-10038-3

57.Iungman T, Cirach M, Marando F, Pereira Barboza E, Khomenko S, Masselot P, et al. Cooling cities through urban green infrastructure: a health impact assessment of European cities. Lancet. 2023;401(10376):577-89. http://doi.org/10.1016/S0140-6736(22)02585-5

Click here to access/download;Figure;Figure 1.jpg

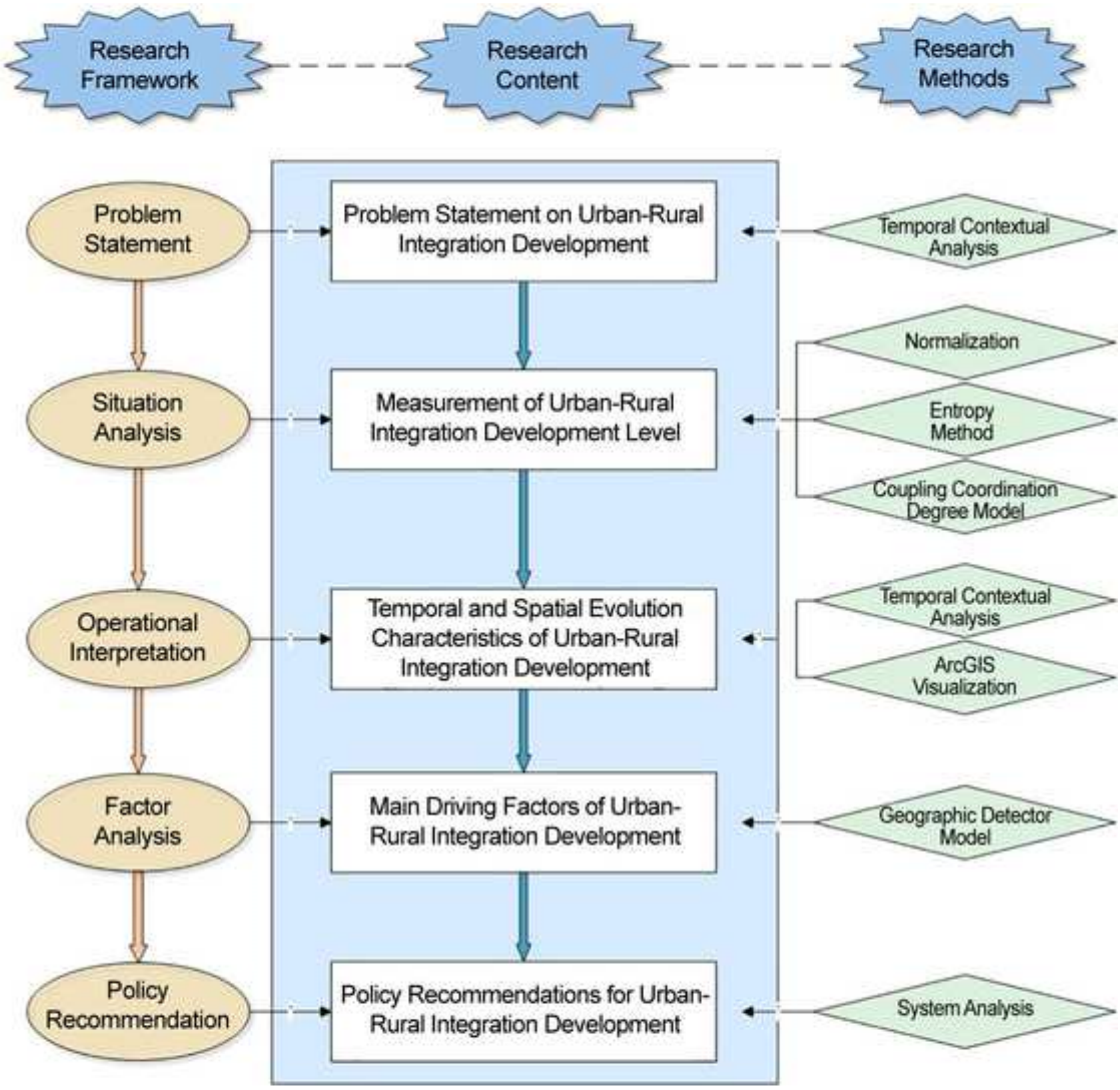

Click here to access/download;Figure;Figure 2.jpg

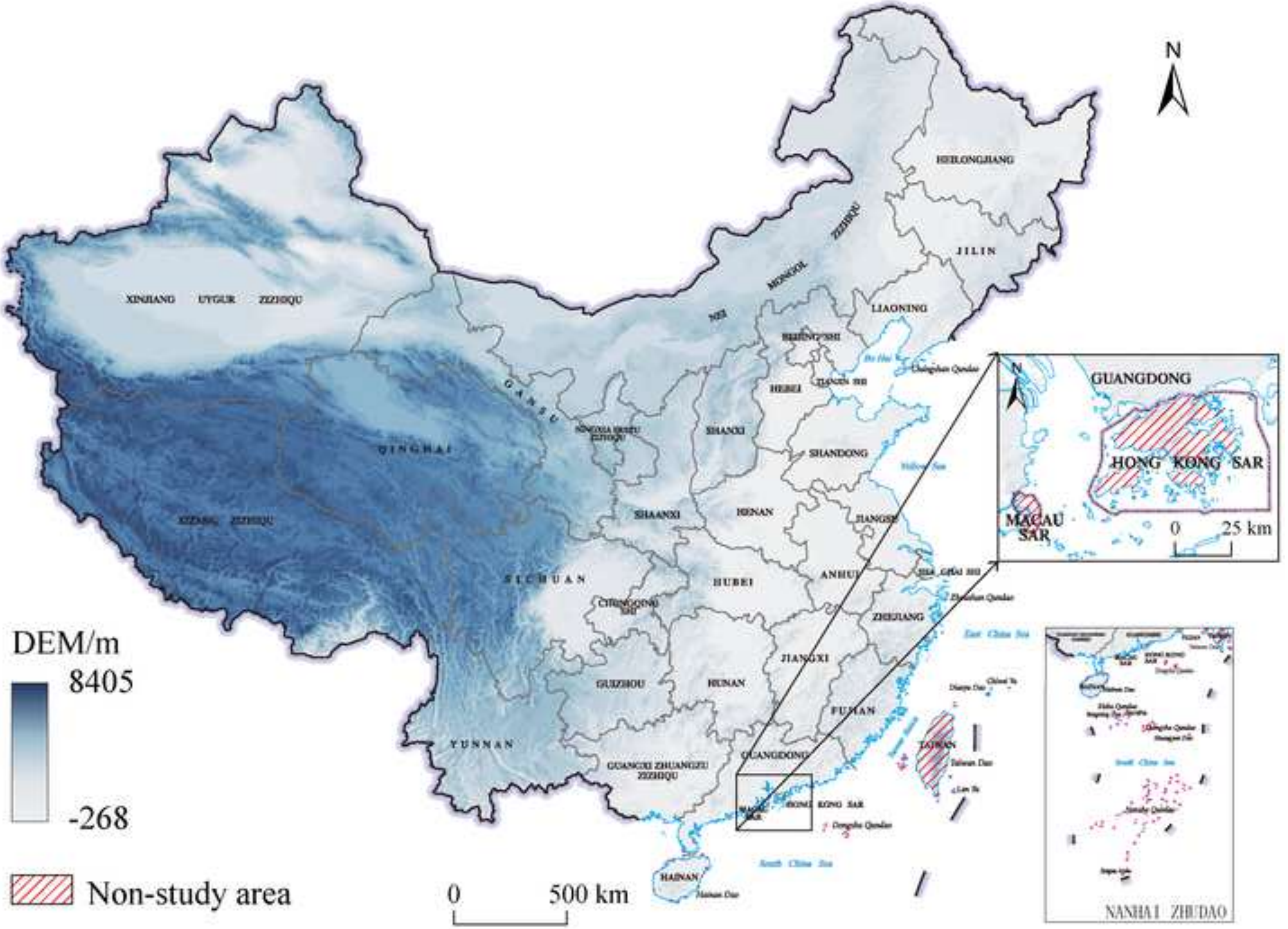

Click here to access/download;Figure;Figure 3.jpg

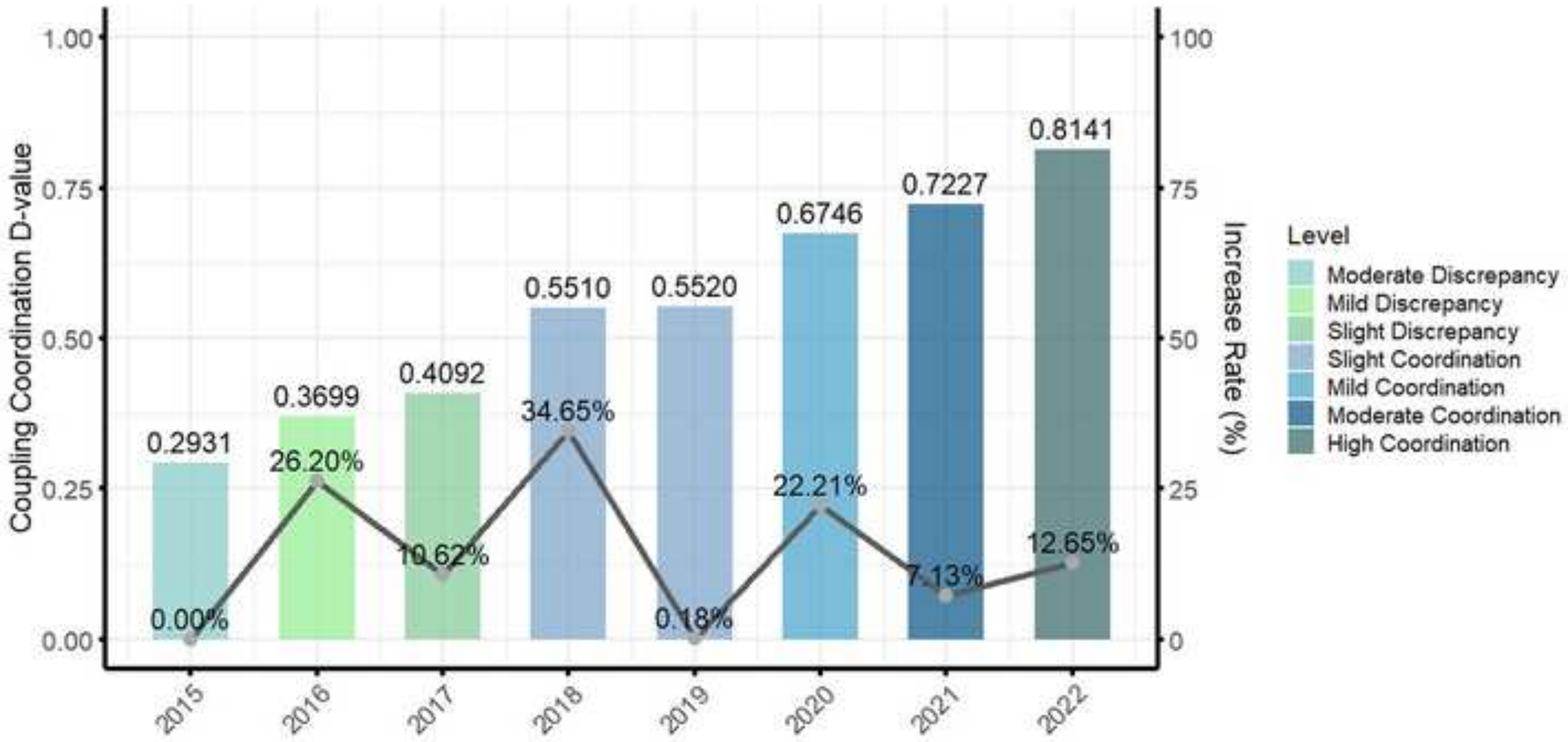

Click here to access/download;Figure;Figure 4.jpg

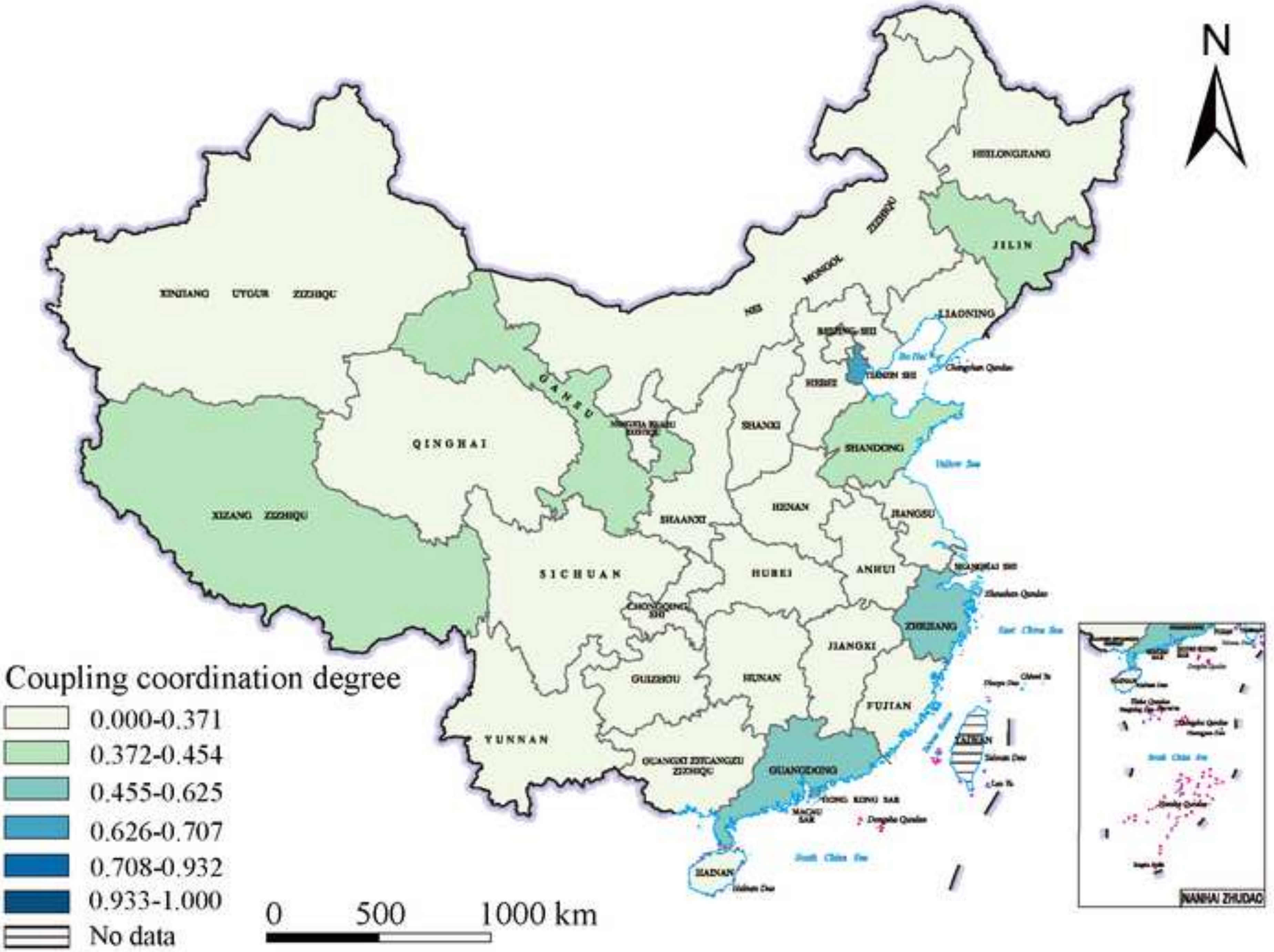

Click here to access/download;Figure;Figure 5.jpg

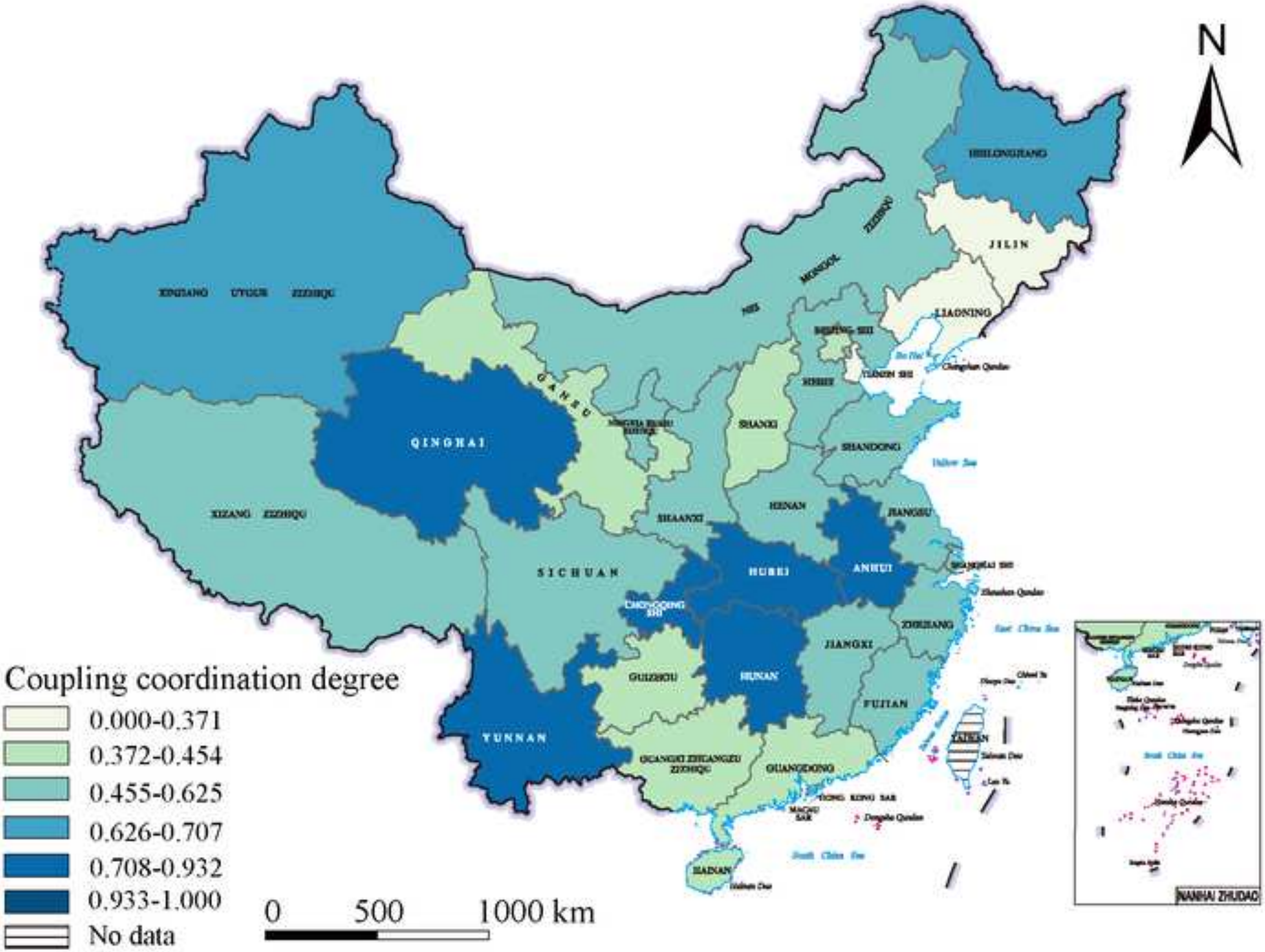

Click here to access/download;Figure;Figure 6.jpg

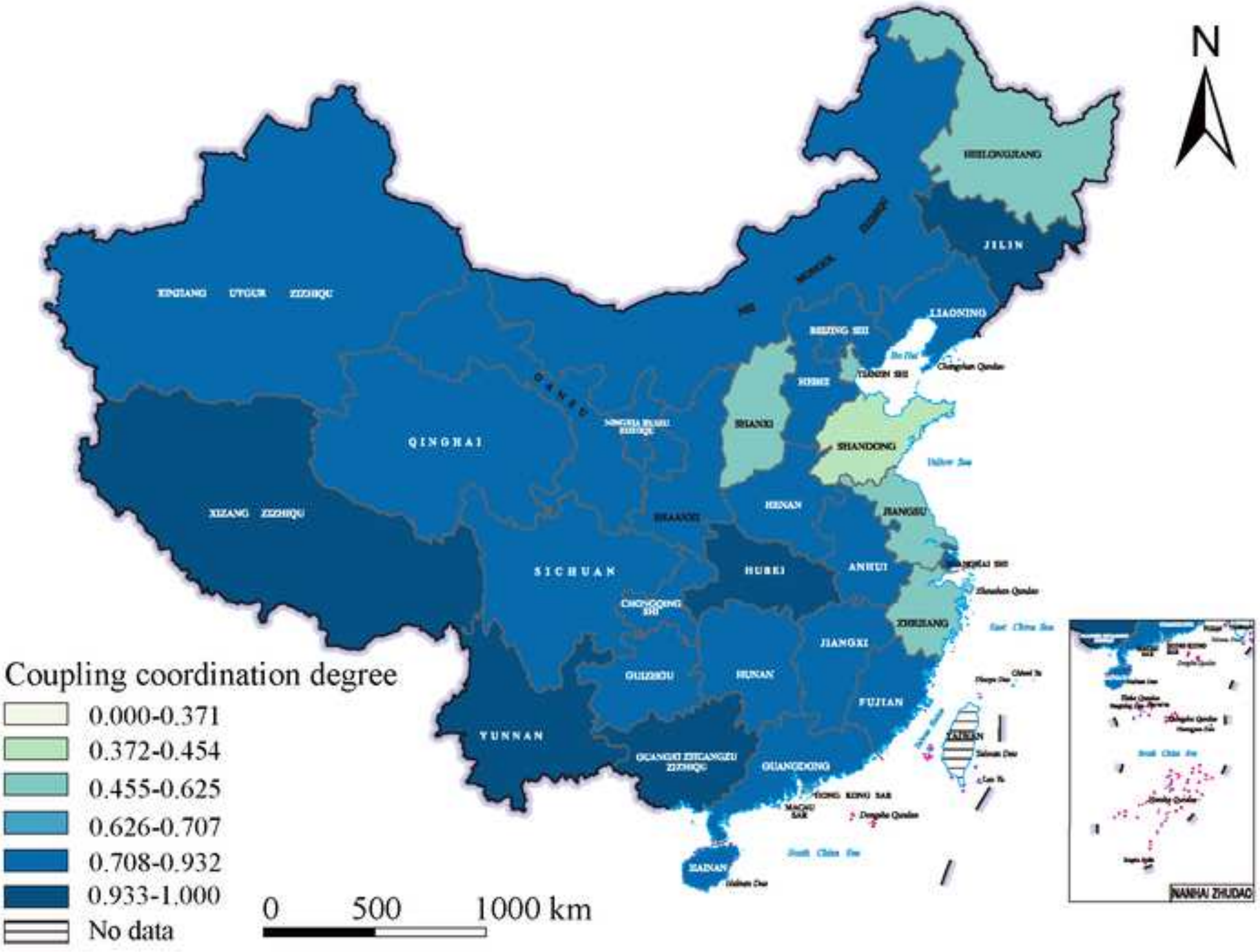

Click here to access/download;Figure;Figure 7.jpg

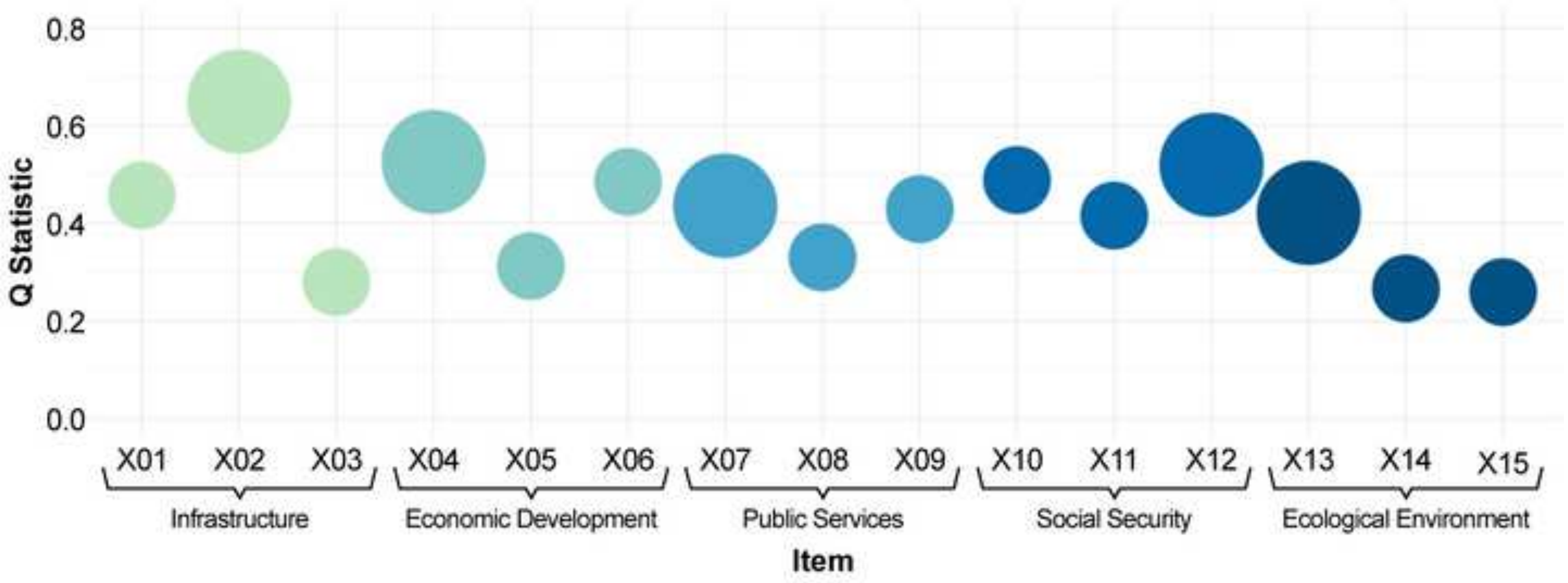

Infrastructure	Q statistic	Economic Development	Q statistic	Public Services	Q statistic	Social Security	Q statistic	Ecological Environment	Q statistic
X01	0.4576	X04	0.5258	X07	0.4360	X10	0.4887	X13	0.4213
X02	0.6498	X05	0.3124	X08	0.3300	X11	0.4158	X14	0.2663
X03	0.2794	X06	0.4850	X09	0.4291	X12	0.5198	X15	0.2589

Click here to access/download
Supporting Information
Supplementary Information.xlsx
