# Supplementary material for: The spatiotemporal evolution and driving factors of urban–rural integration over the past eight years in China: Evidence from 31 provinces
Source: PLoS One. 2025 Dec 2;20(12):e0337317. doi: 10.1371/journal.pone.0337317 (PMC12671771; doi:10.1371/journal.pone.0337317)
Supplement: Attachment — Submitted filename: Response to reviewers.docx [file pone.0337317.s005.docx]

Dear Editor,

It is a great honor to receive your letter. I sincerely thank you and the reviewers for your careful reading and valuable comments on this manuscript. After receiving the feedback, we have thoroughly reviewed each point and made significant revisions to the manuscript accordingly. We now submit the detailed responses and specific modifications for your review. We kindly ask you and the reviewers to review it once again. Thank you!

**Response to Editor’s Comments:**

**Question 1:** Insufficient justification for defining and applying "comparative coefficient" indicators, including ambiguous ideal values and inconsistent directional attributes.

**Answer 1:** Thank you very much for your suggestions! We completely agree that the explanation of the "comparative coefficient" indicator, its ideal value setting, and directional processing in the original manuscript were insufficient. Therefore, we have made comprehensive revisions to the relevant section. The specific explanation is as follows, please review it.

Firstly, regarding the calculation of the comparative coefficient, we would like to clarify that all "comparative coefficients" in the paper are uniformly defined as "urban indicator value / rural indicator value." Taking the "urban-rural road length comparison coefficient" as an example, the calculation formula is: urban road length / rural road length. To avoid ambiguity, we have uniformly indicated the calculation methods for each indicator in the "Indicator Description" column of Table 1 in the paper.

Secondly, the core basis for selecting the comparative coefficient to measure urban-rural integration is that the ideal goal of urban-rural integration is to narrow the urban-rural gap and promote balanced development. The ideal value for such indicators is 1, meaning that urban and rural areas reach complete balance in this indicator. The further the ratio deviates from 1 (whether significantly greater or less than 1), the greater the gap between urban and rural areas, and the lower the degree of integration. Therefore, the comparative coefficient can clearly and intuitively reflect the degree of difference between urban and rural areas, making it suitable for measuring the level of integration.

Next, regarding the selection of the attribute (+/−) for indicators, we would like to explain that the indicator system in this study mainly includes two types: one type is comparative indicators (e.g., "urban-rural road length comparison coefficient"), and the other type is descriptive indicators (e.g., "urban-rural pension insurance coverage rate"). For comparative indicators, the attribute determination is based on the following logic: the degree of deviation from 1 represents the size of the urban-rural gap. The larger the value, the greater the gap and the lower the integration degree, which is negatively related to the integration goal, so we assign a "−" attribute. For descriptive indicators, the attribute determination is based on the fact that these indicators represent the development level of a particular dimension. A higher value indicates better progress in that area and a more significant positive effect on urban-rural integration development, thus they are assigned a "+" attribute.

**Question 2:** Lack of theoretical depth in framing urban-rural theories and inadequate literature engagement, particularly regarding research gaps.

**Answer 2:** Thank you for your valuable comments! We fully agree that the original manuscript had shortcomings in the theoretical analysis of urban-rural relations, the use of literature, and the explanation of research gaps. As a result, we have made significant revisions to the literature review section. The specific content is as follows, please review it.

On this basis, further theoretical doctrines have been developed, including the “Urban Bias Theory” “Rural Bias Theory” “Urban-Rural Dual Structure Theory” and “Urban-Rural Integrated Development Theory”. The Urban Bias Theory advocates for an urban-centered development path, emphasizing that policies and resources should concentrate in urban areas. However, this often leads to the stagnation of rural development and a widening development gap between urban and rural areas. The Rural Bias Theory, in contrast, focuses on the intrinsic dynamics of rural areas, advocating for sustainable progress based on rural resources. However, this theory somewhat neglects the role of urban areas in driving and influencing rural development, and in practice, it often falls into the trap of isolated development. The Urban-Rural Dual Structure Theory focuses on the structural contradictions caused by the coexistence of traditional and modern sectors, revealing how institutional divisions constrain resource flow and social equity. However, its analysis tends to be static, lacking sufficient explanation of the dynamic mechanisms of urban-rural interaction and integration. The Urban-Rural Integrated Development Theory, on the other hand, aims to break down the development barriers between urban and rural areas, advocating for complementary functions, institutional coordination, and systemic integration, promoting two-way flows of elements and resource sharing, and providing a theoretical direction for achieving genuine integrated development.

In summary, existing research on urban-rural integration mainly focuses on theoretical foundations, core concepts, practical exploration, and policy recommendations. These achievements are largely based on normative analytical frameworks, providing important references for the field. However, constrained by traditional research perspectives and analytical frameworks, existing studies still have notable shortcomings in systematically understanding the mechanisms of urban-rural interaction and the integration of multi-dimensional elements. To fill this research gap, this study adopts a holistic perspective on urban-rural integration and constructs a five-dimensional analysis framework that includes "infrastructure—economic development—social services—social security—ecological environment." Within this framework, infrastructure is regarded as the material foundation for the flow of urban and rural elements, while social security serves as an important institutional support for promoting social equity. Together, these dimensions provide a new analytical perspective for understanding urban-rural integration. Based on panel data from 31 provinces in China from 2015 to 2022, this study employs methods such as the entropy weight method, coupling coordination degree model, and geographic detector to systematically examine the national level of urban-rural integration, its temporal and spatial evolution characteristics, and key driving mechanisms. The aim is to provide more comprehensive theoretical support and empirical references for achieving coordinated urban-rural development.

**Question 3:** Unexplained data volatility (e.g., abrupt drops in provincial integration indices) and methodological inconsistencies in weighting sub-systems.

**Answer 3:** Thank you very much for your valuable suggestions! We fully agree that the original manuscript’s explanation of abnormal data fluctuations and the description of the subsystem weighting method were indeed insufficient. We have now added supplementary analysis and explanations to the relevant sections. The specific revisions are as follows, please review them.

Economic structural adjustment is the key driving force behind the "fluctuating upward" trend in urban-rural integration development. Alongside market demand contraction and the widespread adoption of digital technologies, traditional heavy industries and the energy sector are generally facing declining efficiency and shrinking employment, which has, to some extent, exacerbated the income gap between urban and rural areas, creating phase-specific pressures on the integration process. At the same time, emerging business models such as leisure agriculture and rural tourism have flourished, not only driving the continued expansion of e-commerce for agricultural products but also accelerating the influx of social capital into new rural sectors. These new industries effectively absorb the production factors released by the transformation of traditional sectors, creating a large number of new employment opportunities in rural areas, significantly expanding farmers' income channels, and injecting sustained and powerful endogenous momentum into urban-rural integration. In this transformation process, there is a clear dynamic tug-of-war between the decline of traditional industries and the rise of emerging industries: the short-term pain caused by the adjustment of traditional sectors intertwines with the long-term benefits brought by the expansion of the new economy. However, as the growth potential and driving effects of the new driving forces continue to strengthen, their upward momentum generally surpasses the downward pressure brought by the decline of traditional sectors, thus driving the overall "fluctuating upward" development trend of national urban-rural integration. Compared to existing research, this paper not only verifies the profound impact of economic structural adjustments on urban-rural integration development, as proposed by Xu, but also deepens the understanding of the structural causes behind the "fluctuating upward" trend in urban-rural integration from the perspective of the transition between old and new driving forces.

The comprehensive evaluation system constructed in this study includes two levels of weights: within the system and between systems. At the internal level of the system, the entropy weight method is used to objectively determine the weights of each indicator; at the inter-system level, the β weight reflects the relative importance of each subsystem (e.g., infrastructure, economic development, etc.) within the overall urban-rural integration theoretical framework. These two levels of weights address different issues and together form a complete two-stage evaluation model. Therefore, there is no inconsistency at the methodological level.

**Question 4:** Superficial discussion of findings, policy implications, and driving mechanisms (e.g., broadband internet’s role).

**Answer 4:** Thank you for your valuable comments! We fully agree that the original manuscript had shortcomings in discussing the research results and providing policy recommendations. It did not deeply analyze the causes of the results and offer specific suggestions. Therefore, we have made significant revisions to the discussion and policy recommendations sections. The specific content is as follows, please review it.

**Discussion**

**Analysis of temporal evolution characteristics**

The overall development of urban-rural integration in China is on the rise, but the growth rate is uneven, displaying a wave-like evolution pattern of "rapid growth-slow development-fluctuating rise." The reasons behind this phenomenon are analyzed as follows: First, the release of policy dividends is the core driving force behind the rapid development of urban-rural integration. The country has introduced a series of top-level design documents such as the "Opinions on Establishing and Improving the System and Policy Framework for Urban-Rural Integration," with breakthroughs in land system reforms (such as allowing rural collective commercial construction land to enter the market) and household registration system reforms (such as fully lifting urban-rural household registration restrictions). These reforms have broken long-standing institutional barriers that constrained the free flow of resources. This loosening of regulations has significantly stimulated market vitality. By 2018, the rural land transfer rate reached 36.79%, an increase of 21.18% compared to before the policy was implemented. Meanwhile, the number of migrant workers returning to rural areas to start businesses reached 7.8 million, a 62.5% increase compared to before the policy was implemented, effectively activating the endogenous driving force of rural development. On this basis, the policy has focused on increasing fiscal transfer payments to address gaps in rural public services. In the field of education, in 2018, the per-student educational expenditure for rural middle school students was 13,912 yuan, an increase of 41.2% compared to before the policy was implemented. Meanwhile, the rate of rural participation in the new rural cooperative medical insurance reached 99.45%, an increase of 0.56% compared to before the policy was implemented, narrowing the gap in basic public services between urban and rural areas and providing solid livelihood guarantees for urban-rural integration. Furthermore, the policy has focused on promoting the deep integration of urban and rural industries. By cultivating modern agricultural industrial parks, building characteristic towns, and other platforms, it has guided urban capital and technology to flow into rural areas, integrating them with rural resource endowments and ecological advantages. By 2018, the added value of rural secondary and tertiary industries accounted for 92.8% of GDP, an increase of 2.3% compared to before the policy was implemented, gradually forming a new pattern of linked industrial development between urban and rural areas. Compared to existing studies, this research not only validates the role of policies proposed by Ma in promoting urban-rural integration, but also reveals that the policies mainly promote rapid urban-rural integration through a four-dimensional mechanism of "institutional breaking-resource activation-service balancing-industry linkage."

However, after entering 2019, the outbreak of the COVID-19 pandemic became a key obstacle to the slow development of urban-rural integration. In order to prevent the spread of the virus, the government implemented strict transportation controls and restrictions on interregional movement, which temporarily hindered the normal flow of production factors between urban and rural areas. By 2020, the number of migrant workers leaving for work was only 169.59 million, a decrease of 2.67% compared to the previous year. Meanwhile, the agricultural product logistics system was inefficient, leading to a disconnection between regional production and sales of fresh agricultural products. In some major agricultural production counties, the unsold rate exceeded 25%, significantly weakening the urban-rural economic cycle and causing a temporary decline in rural household incomes. Against this backdrop, emerging rural businesses relying on labor mobility and scene-based consumption were particularly hard-hit. By 2020, rural tourism revenue amounted to 600 billion yuan, a 66.9% decrease compared to the previous year. A large number of small businesses and individual operators, dependent on cultural and tourism services, faced existential crises. Compared to existing studies, this research not only identifies the pandemic as an exogenous shock, as emphasized by Li, but also further reveals that its impact mainly slowed down the urban-rural integration process through three core aspects: "disrupting the flow of resources—weakening the urban-rural economic cycle—impacting the rural industrial ecosystem."

Economic structural adjustment is the key driving force behind the "fluctuating upward" trend in urban-rural integration development. Alongside market demand contraction and the widespread adoption of digital technologies, traditional heavy industries and the energy sector are generally facing declining efficiency and shrinking employment, which has, to some extent, exacerbated the income gap between urban and rural areas, creating phase-specific pressures on the integration process. At the same time, emerging business models such as leisure agriculture and rural tourism have flourished, not only driving the continued expansion of e-commerce for agricultural products but also accelerating the influx of social capital into new rural sectors. These new industries effectively absorb the production factors released by the transformation of traditional sectors, creating a large number of new employment opportunities in rural areas, significantly expanding farmers' income channels, and injecting sustained and powerful endogenous momentum into urban-rural integration. In this transformation process, there is a clear dynamic tug-of-war between the decline of traditional industries and the rise of emerging industries: the short-term pain caused by the adjustment of traditional sectors intertwines with the long-term benefits brought by the expansion of the new economy. However, as the growth potential and driving effects of the new driving forces continue to strengthen, their upward momentum generally surpasses the downward pressure brought by the decline of traditional sectors, thus driving the overall "fluctuating upward" development trend of national urban-rural integration. Compared to existing research, this paper not only verifies the profound impact of economic structural adjustments on urban-rural integration development, as proposed by Xu, but also deepens the understanding of the structural causes behind the "fluctuating upward" trend in urban-rural integration from the perspective of the transition between old and new driving forces.

**Analysis of spatial distribution characteristics**

The urban-rural integration development in China shows distinct spatial heterogeneity, forming a diverse development pattern where the eastern coastal region leads, the central region rises rapidly, and the western region has great potential. This spatial structure is closely related to the basic conditions and development paths of different regions. The eastern coastal regions, benefiting from superior geographical locations, have actively developed an open economy, gradually building a solid economic foundation, attracting large amounts of capital and high-end industries, and effectively promoting the early development of urban-rural integration through industrial gradient transfer mechanisms. Taking Zhejiang Province as an example, this province is located on the southeast coast and is home to the Ningbo-Zhoushan Port, the largest cargo throughput port in the world. Its unique geographical advantages provide a solid foundation for the development of an open economy. Leveraging this condition, Zhejiang has vigorously developed port industries and cross-border e-commerce, gradually building a strong economic base. By 2022, Zhejiang’s per capita GDP reached 118,496 yuan, ranking fourth among all provinces and regions in China. On this basis, the province has attracted a large amount of capital and high-end industries by improving infrastructure and continuously optimizing the business environment. However, as the costs of land, labor, and other factors in the central cities continue to rise, many high-value-added industries such as export processing and smart manufacturing have gradually spread to counties and rural areas with larger development spaces and lower overall costs. This process has significantly driven industrial upgrading and expanded employment opportunities in rural areas, effectively narrowing the urban-rural development gap. By 2022, the urban-rural income ratio in Zhejiang had dropped to 1.90, significantly lower than the national average, making it one of the provinces with the most significant achievements in urban-rural coordinated development. As pointed out by Wang's research, the economic development level plays a key role in urban-rural integration. However, this study further emphasizes that urban-rural integration not only depends on the overall economic level but is also closely related to the restructuring of industrial space, actively promoting urban-rural integration towards a higher level of development.

The central region, benefiting from its abundant labor resources, has effectively aligned with the market demands of the eastern coastal regions. An increasing number of labor-intensive industries such as textiles and apparel, electronics, and home appliance manufacturing have chosen to relocate to the central region, where factors like land and labor costs have a comparative advantage. In this process, large amounts of capital, technology, and management expertise have continuously flowed into the central region through direct investment, industrial transfer, and regional cooperation, not only driving the transformation and upgrading of local traditional industries but also accelerating the agglomeration of emerging industries. It is particularly noteworthy that these industrial transfers have not only remained at the city level but have shown significant "county-oriented" characteristics. Taking Henan Province as an example, the province has actively taken on industrial transfer from the Yangtze River Delta region by building modern industrial clusters, creating several specialized industrial clusters at the county level. For example, the textile and apparel industrial cluster in Taikang County, Zhoukou City, not only attracted well-known enterprises from the eastern region but also extended the industrial chain to rural areas through a "leading enterprise + rural workshop" model, directly providing employment and income growth for nearly 100,000 rural laborers. The smart sensor industrial cluster formed in Nanyang City, in addition to setting up core research and manufacturing links around the city, has also dispersed supporting links such as packaging, logistics, and basic component production to surrounding towns, promoting the deep integration and collaborative development of urban and rural industrial chains. As pointed out by Ma's research, industrial transfer has injected strong momentum into urban-rural integration. However, this study further emphasizes that the deep embedding of industries at the county and rural levels not only promotes the reallocation of production factors between urban and rural areas but also significantly drives urban-rural integration towards higher quality development through job creation and industrial chain collaboration.

In contrast, the western region faces more prominent urban-rural dual structure issues due to its inland location and complex ecological environment. However, with the continuous support of national strategies such as the Western Development Program, poverty alleviation, and rural revitalization, the region has made significant progress in breaking through the bottleneck of urban-rural integration. Taking Sichuan Province as an example, its "characteristic agriculture + rural tourism" dual-driven model in the Qinba Mountains and Liangshan Yi Autonomous Prefecture has not only activated rural industries but has also become an important mechanism for connecting urban and rural areas and promoting integration. On one hand, by developing specialty industries such as highland agriculture and ecological breeding, and leveraging ethnic culture and ecological resources to promote rural tourism, rural areas have gradually established high-value-added industrial systems, strengthening their connections with urban consumer markets. On the other hand, the opening of e-commerce channels has not only achieved the smooth flow of both "agricultural products going up" and "industrial products going down," but has also profoundly transformed the traditional urban-rural exchange model, promoting the more fair and efficient flow of resources and elements between urban and rural areas. These measures have not only promoted income growth for farmers and diversified rural economies but have also fundamentally reshaped urban-rural relations. Rural areas are no longer a simple resource exporter or a passive recipient of development but are gradually growing into development subjects that complement urban functions and share value. In this process, characteristic industries have become the link for urban-rural industrial collaboration, e-commerce platforms have become bridges for connecting urban and rural markets, and policy guidance has provided a solid guarantee for institutional integration between urban and rural areas. As pointed out by Wang's research, policy support plays a key role in driving urban-rural integration. However, this study further emphasizes that the integration of urban and rural areas in the western region not only depends on external resource inputs but, more fundamentally, on the construction of endogenous driving mechanisms. Only by fully unleashing the local development potential of rural areas can urban-rural integration move towards a more sustainable direction.

**Analysis of driving factors**

The development of urban-rural integration in China is influenced by multiple factors, including infrastructure, economic development, public services, social security, and the ecological environment. Among these, broadband internet access, per capita disposable income, education expenditure, unemployment insurance coverage, and green coverage rate have played key roles in promoting this development.

Specifically, broadband internet access is the cornerstone of urban-rural information resource sharing, directly impacting the speed of information transmission, knowledge acquisition, and cultural exchange. However, due to underdeveloped infrastructure, the broadband access rate in rural areas is generally lower than in urban areas, leading to a significant digital divide. Therefore, reducing the gap in broadband access between urban and rural areas is crucial for achieving information sharing and further promoting urban-rural integration.

Per capita disposable income, as an important indicator of economic development, directly reflects the consumption capacity of urban and rural residents. However, due to the simple industrial structure and backward production technology in rural areas, per capita disposable income is generally lower than in urban areas, which continues to widen the income gap between urban and rural areas. This income disparity not only lowers the consumption capacity of rural residents but also hinders the overall economic development of rural areas. Therefore, narrowing the gap in per capita disposable income between urban and rural areas is of paramount importance for improving the level of economic development and promoting urban-rural integration.

Education expenditure is a key means of improving the quality of human capital in urban and rural areas. However, rural areas currently face a shortage of educational investment, primarily due to the low income levels of rural families, which makes it difficult for them to bear high educational costs. This disparity in educational resources not only exacerbates the inequality in educational opportunities between urban and rural residents but also severely hinders the overall improvement of human capital quality in rural areas. Therefore, reducing the gap in education expenditure between urban and rural areas is crucial for enhancing human capital quality and promoting urban-rural integration.

Unemployment insurance coverage is an important indicator of the completeness of the social security system in urban and rural areas. However, due to inadequate institutional design, the unemployment insurance coverage rate in rural areas is generally lower than in urban areas. This disparity not only leaves rural residents inadequately protected against the risk of unemployment but also limits the economic development of rural areas. Therefore, narrowing the gap in unemployment insurance coverage between urban and rural residents is crucial for improving the social security system and promoting urban-rural integration.

Green coverage rate is an important indicator of regional ecological environmental quality. However, due to economic advantages and policy support, urban areas generally have a higher green coverage rate than rural areas. This disparity not only results in unequal access to green ecological benefits for urban and rural residents but also hinders the improvement of the ecological environment and quality of life in rural areas. Therefore, narrowing the gap in green coverage rate between urban and rural areas is crucial for improving regional ecological environmental quality and promoting urban-rural integration.

**Recommendations**

In light of the development characteristics of urban-rural integration in different stages in China, it is necessary to adopt targeted policy measures. During the rapid growth stage, the fundamental contradiction faced by urban-rural integration is the disconnect between traditional institutional frameworks and development needs. At this point, policies should focus on institutional breakthroughs and infrastructure construction to inject strong initial momentum into the integration process. Specifically, efforts should be made to vigorously promote rural land system reforms and the market-oriented allocation of factors, eliminating institutional barriers that hinder resource flow. At the same time, the construction of county-level industrial parks and digital infrastructure should be accelerated, actively attracting industrial spillovers from central cities. Systematic vocational skills training should be introduced to enhance the rural labor force's ability to migrate and find employment, laying a solid foundation for urban-rural integration. When the development stage shifts to slower growth, the extensive model of relying solely on factor input is no longer sustainable, and urban-rural integration urgently needs to transition from scale expansion to quality improvement. During this phase, policy focus should shift to fostering endogenous development momentum. On one hand, efforts should be made to promote the equalization of public services such as education and healthcare, reducing the social gap between urban and rural areas and creating a favorable environment for high-quality development. On the other hand, policies should actively cultivate emerging sectors such as rural tourism and e-commerce, encouraging rural industrial diversification and achieving a critical shift from "external support" to "self-sustaining growth." In the fluctuation and rising stage, the main challenge of urban-rural integration lies in insufficient system stability and weak risk resistance. Policies must focus on building risk buffering mechanisms and stabilizing development expectations, with two key aspects: First, establishing a dynamic monitoring and early warning system for urban-rural integration to promptly identify socio-economic risks, and innovatively using fiscal and financial tools for counter-cyclical regulation. For instance, rural infrastructure construction projects can be initiated at appropriate times to stabilize livelihoods during periods of increased employment pressure. Second, maintaining policy continuity and stability, strengthening government credibility, and providing market players with a predictable institutional environment to fundamentally enhance the sustainability of urban-rural integration.

Faced with the development characteristics of urban-rural integration in different regions of China, differentiated development strategies should be adopted. In the eastern coastal regions, efforts should focus on promoting the aggregation of high-end factors and breakthrough innovations, with an emphasis on developing smart agriculture, digital rural areas, and future communities. At the same time, traditional manufacturing industries should be guided to move in an orderly manner to the central and western regions, and key institutional barriers, such as land and household registration, should be overcome to provide replicable and scalable practical models for urban-rural integration across the country. In the central regions, there is a need to strengthen industrial undertaking and hub functions, accelerate the improvement of transportation logistics infrastructure and business environments, actively undertake industrial transfers from the east, cultivate county-level characteristic industrial clusters, explore the construction of "remote industrial zones" with eastern provinces, deepen cross-provincial industrial cooperation and talent exchange, and enhance regional collaborative development capabilities. In the western regions, central government fiscal support should play a leading role in speeding up the improvement of infrastructure and public service gaps. At the same time, leveraging ecological resource advantages, the focus should be on cultivating green industries such as ecological agriculture, clean energy, and cultural tourism, and creating a distinctive and sustainable integration development path.

In light of the various factors influencing urban-rural integration in China, it is necessary to focus on key influencing factors to achieve breakthroughs. In terms of improving internet broadband access, the "Broadband Rural" initiative should be continuously promoted, along with subsidy policies for service fees, with the goal of achieving full coverage of gigabit optical networks in administrative villages by 2025, significantly improving the penetration rate of 100Mbps broadband in rural households. At the same time, digital service centers at the county level should be established, strengthening technical training and application guidance to ensure that broadband networks not only provide good connectivity but also deliver tangible benefits. In terms of increasing rural residents' per capita disposable income, the "One Township, One Product" income enhancement plan should be implemented, with each township focusing on cultivating 1-2 deep processing projects for characteristic agricultural products. Additionally, a rural e-commerce direct-to-market system should be established, setting up standardized service centers for agricultural products at the county and township levels, integrating packaging, testing, logistics, and other elements, and providing one-stop service support. In optimizing educational expenditure, the "County-Managed, School-Employed" teacher management reform should be deepened to ensure the benefits of rural teachers. At the same time, a pairing mechanism between urban and rural schools should be established, promoting the extension of high-quality educational resources to rural areas, thus facilitating the balanced development of education between urban and rural areas. In expanding the coverage of unemployment insurance, migrant workers and workers in new employment forms should be fully included in the security system, with a combined model of "government subsidies + enterprise contributions + voluntary individual participation" for insurance enrollment. At the same time, through the nationwide unified social security service platform, the seamless transfer and continuation of unemployment insurance relationships across regions should be realized, effectively enhancing the ability to cope with unemployment risks. In terms of improving urban-rural greening coverage, the rural greening project should be steadily promoted with annual growth targets. Meanwhile, a systematic approach to urban-rural ecological restoration should be carried out to continuously improve living conditions in both urban and rural areas, enhancing the quality of the ecological environment.

**Question 5:** Low-resolution figures and non-intuitive data presentation (Table 3).

**Answer 5:** Thank you for your valuable comments! We fully agree that the quality of the charts and the presentation of data in the original manuscript were indeed inadequate, which caused inconvenience for readers’ understanding. As a result, we have thoroughly updated all illustrations in the manuscript (including Figures 1 to 7), using high-resolution formats (300 DPI and above) to improve image clarity and publishing professionalism.

Regarding the data presentation issue in Table 3, we strongly agree that using charts would provide a more intuitive way to present the spatiotemporal evolution characteristics of urban-rural integration development. In fact, in this study, we have already created Figures 3 to 6 based on the data in Table 3, and systematically analyzed the temporal dynamics and spatial patterns of urban-rural integration development levels at both national and regional levels. The main purpose of retaining Table 3 is to provide readers with the complete original calculated values, so they can verify or explore the data in more depth. The core analysis of trends and regional comparisons in the text is primarily centered around the corresponding charts. We sincerely hope that this original design will be understood. Your support is of utmost importance to us.

**Response to Reviewer 1’s Comments:**

**Question 1:** It would be interesting if the article provided more specific information about the findings, such as concrete examples of the regions compared and their specificities, thus making it clearer how public policies could be implemented to improve the performance evaluated.

**Answer 1:** Thank you very much for your valuable suggestion! We completely agree that providing specific case studies for different regions and more clearly outlining the details of policy implementation will help enhance the practical value of the research. Based on your feedback, we have added the relevant content in the sections "Analysis of spatial distribution characteristics".The specific additions are as follows.

**Analysis of spatial distribution characteristics**

The urban-rural integration development in China shows distinct spatial heterogeneity, forming a diverse development pattern where the eastern coastal region leads, the central region rises rapidly, and the western region has great potential. This spatial structure is closely related to the basic conditions and development paths of different regions. The eastern coastal regions, benefiting from superior geographical locations, have actively developed an open economy, gradually building a solid economic foundation, attracting large amounts of capital and high-end industries, and effectively promoting the early development of urban-rural integration through industrial gradient transfer mechanisms. Taking Zhejiang Province as an example, this province is located on the southeast coast and is home to the Ningbo-Zhoushan Port, the largest cargo throughput port in the world. Its unique geographical advantages provide a solid foundation for the development of an open economy. Leveraging this condition, Zhejiang has vigorously developed port industries and cross-border e-commerce, gradually building a strong economic base. By 2022, Zhejiang’s per capita GDP reached 118,496 yuan, ranking fourth among all provinces and regions in China. On this basis, the province has attracted a large amount of capital and high-end industries by improving infrastructure and continuously optimizing the business environment. However, as the costs of land, labor, and other factors in the central cities continue to rise, many high-value-added industries such as export processing and smart manufacturing have gradually spread to counties and rural areas with larger development spaces and lower overall costs. This process has significantly driven industrial upgrading and expanded employment opportunities in rural areas, effectively narrowing the urban-rural development gap. By 2022, the urban-rural income ratio in Zhejiang had dropped to 1.90, significantly lower than the national average, making it one of the provinces with the most significant achievements in urban-rural coordinated development. As pointed out by Wang**'s** research, the economic development level plays a key role in urban-rural integration. However, this study further emphasizes that urban-rural integration not only depends on the overall economic level but is also closely related to the restructuring of industrial space, actively promoting urban-rural integration towards a higher level of development.

The central region, benefiting from its abundant labor resources, has effectively aligned with the market demands of the eastern coastal regions. An increasing number of labor-intensive industries such as textiles and apparel, electronics, and home appliance manufacturing have chosen to relocate to the central region, where factors like land and labor costs have a comparative advantage. In this process, large amounts of capital, technology, and management expertise have continuously flowed into the central region through direct investment, industrial transfer, and regional cooperation, not only driving the transformation and upgrading of local traditional industries but also accelerating the agglomeration of emerging industries. It is particularly noteworthy that these industrial transfers have not only remained at the city level but have shown significant "county-oriented" characteristics. Taking Henan Province as an example, the province has actively taken on industrial transfer from the Yangtze River Delta region by building modern industrial clusters, creating several specialized industrial clusters at the county level. For example, the textile and apparel industrial cluster in Taikang County, Zhoukou City, not only attracted well-known enterprises from the eastern region but also extended the industrial chain to rural areas through a "leading enterprise + rural workshop" model, directly providing employment and income growth for nearly 100,000 rural laborers. The smart sensor industrial cluster formed in Nanyang City, in addition to setting up core research and manufacturing links around the city, has also dispersed supporting links such as packaging, logistics, and basic component production to surrounding towns, promoting the deep integration and collaborative development of urban and rural industrial chains. As pointed out by Ma**'s** research, industrial transfer has injected strong momentum into urban-rural integration. However, this study further emphasizes that the deep embedding of industries at the county and rural levels not only promotes the reallocation of production factors between urban and rural areas but also significantly drives urban-rural integration towards higher quality development through job creation and industrial chain collaboration.

In contrast, the western region faces more prominent urban-rural dual structure issues due to its inland location and complex ecological environment. However, with the continuous support of national strategies such as the Western Development Program, poverty alleviation, and rural revitalization, the region has made significant progress in breaking through the bottleneck of urban-rural integration. Taking Sichuan Province as an example, its "characteristic agriculture + rural tourism" dual-driven model in the Qinba Mountains and Liangshan Yi Autonomous Prefecture has not only activated rural industries but has also become an important mechanism for connecting urban and rural areas and promoting integration. On one hand, by developing specialty industries such as highland agriculture and ecological breeding, and leveraging ethnic culture and ecological resources to promote rural tourism, rural areas have gradually established high-value-added industrial systems, strengthening their connections with urban consumer markets. On the other hand, the opening of e-commerce channels has not only achieved the smooth flow of both "agricultural products going up" and "industrial products going down," but has also profoundly transformed the traditional urban-rural exchange model, promoting the more fair and efficient flow of resources and elements between urban and rural areas. These measures have not only promoted income growth for farmers and diversified rural economies but have also fundamentally reshaped urban-rural relations. Rural areas are no longer a simple resource exporter or a passive recipient of development but are gradually growing into development subjects that complement urban functions and share value. In this process, characteristic industries have become the link for urban-rural industrial collaboration, e-commerce platforms have become bridges for connecting urban and rural markets, and policy guidance has provided a solid guarantee for institutional integration between urban and rural areas. As pointed out by Wang**'s** research, policy support plays a key role in driving urban-rural integration. However, this study further emphasizes that the integration of urban and rural areas in the western region not only depends on external resource inputs but, more fundamentally, on the construction of endogenous driving mechanisms. Only by fully unleashing the local development potential of rural areas can urban-rural integration move towards a more sustainable direction.

**Response to Reviewer 2’s Comments:**

**Question 1:** The introduction effectively sets the stage by discussing the evolution of urban-rural relations and the policy context of urban-rural integration in China. However, the description of existing research gaps could be more specific. For instance, beyond stating the lack of “comprehensive national-level study” and content concentration on “traditional dimensions,” it could further explain how these gaps hinder a comprehensive understanding of the complexity of urban-rural integration and how this study’s “five-dimensional analytical framework” addresses these deficiencies. Emphasize the unique theoretical and practical value of this research, for example, how it deepens the understanding of urban-rural factor flow and balanced public resource allocation based on existing literature. Consider citing more official documents or authoritative interpretations of China’s new-era urban-rural development strategies, such as in-depth research on the “Rural Revitalization Strategy”, It is recommended that the references include citations of literature, such as mentioned by Ma et al., (2025) (https://doi.org/10.1371/journal.pone.0313125)、Yu et al., (2025) (https://doi.org/10.1038/s41598-025-08492-3)、Yang et al., (2021) (https://doi.org/10.1016/j.habitatint.2021.102420).

**Answer 1:** Thank you very much for your suggestions! Based on your advice, we have improved the original introduction. First, we have more clearly outlined the main limitations of existing research and explained how this study addresses these limitations. Second, we have further clarified the theoretical and practical value of this research. Finally, we have added several authoritative references you mentioned, providing stronger academic support for the study. The specific content is as follows.

Although urban-rural integration has become an important strategic direction for national development, most studies, con-strained by the traditional urban-rural dual analysis framework, have failed to reveal the intrinsic mechanisms of urban-rural integration from a holistic perspective, limiting a deeper understanding of the dynamic relationship between urban and rural areas. At the same time, existing literature tends to focus on traditional dimensions such as the economy, society, and environment, with insufficient attention given to key dimensions such as infrastructure connectivity and social security. This has made it difficult to fully explain the multi-dimensional driving pathways of urban-rural integration. To address these issues, this study adopts an ur-ban-rural integration perspective and constructs a five-dimensional integrated analysis framework, including "infrastructure—economic development—social services—social security—ecological environment," filling the gap in existing research. This framework not only contributes theoretically to deepening the understanding of the multi-dimensional collaborative mechanisms of urban-rural integration, but also provides scientific, reasonable, and efficient empirical evidence for formulating differentiated urban-rural integration policies at the practical level.

**Question 2:** The literature review traces the conceptual origins of urban-rural integration and outlines the dimensions, methods, and scope of existing research, identifying gaps. However, the theoretical framework discussion could be more in-depth. For example, when mentioning “Urban Bias Theory,” “Rural Bias Theory,” “Urban-Rural Dual Structure Theory,” and “Urban-Rural Integrated Development Theory,” briefly elaborate on their core tenets and contributions to understanding urban-rural relations, explaining how this study extends or engages with them. Furthermore, when pointing out the absence of “infrastructure” and “social security” dimensions in existing research, elaborate on why these two dimensions are crucial for understanding urban-rural integration and connect them with other existing theories or empirical findings. For the “Urban-Rural Dual Structure Theory,” cite its classic work to strengthen the theoretical foundation. Such as Lewis, W. A. (1954). Economic development with unlimited supplies of labour. The Manchester School, 22(2), 139-191.

**Answer 2:** Thank you very much for your suggestions! Based on your advice, we have improved the original literature review. First, we systematically explained the core ideas of the "Urban Bias Theory," "Rural Bias Theory," "Urban-Rural Dual Structure Theory," and "Urban-Rural Integrated Development Theory." Second, we focused on incorporating "infrastructure" and "social security" into the analysis dimensions, emphasizing their important role in promoting urban-rural integration development. Finally, we added the classic works you mentioned as references to further strengthen the theoretical foundation of the study. The specific content is as follows. Please see the following.

On this basis, further theoretical doctrines have been developed, including the “Urban Bias Theory” “Rural Bias Theory” “Urban-Rural Dual Structure Theory” and “Urban-Rural Integrated Development Theory”. The Urban Bias Theory advocates for an urban-centered development path, emphasizing that policies and resources should concentrate in urban areas. However, this often leads to the stagnation of rural development and a widening development gap between urban and rural areas. The Rural Bias Theory, in contrast, focuses on the intrinsic dynamics of rural areas, advocating for sustainable progress based on rural resources. However, this theory somewhat neglects the role of urban areas in driving and influencing rural development, and in practice, it often falls into the trap of isolated development. The Urban-Rural Dual Structure Theory focuses on the structural contradictions caused by the coexistence of traditional and modern sectors, revealing how institutional divisions constrain resource flow and social equity. However, its analysis tends to be static, lacking sufficient explanation of the dynamic mechanisms of urban-rural interaction and integration. The Urban-Rural Integrated Development Theory, on the other hand, aims to break down the development barriers between urban and rural areas, advocating for complementary functions, institutional coordination, and systemic integration, promoting two-way flows of elements and resource sharing, and providing a theoretical direction for achieving genuine integrated development.

In summary, existing research on urban-rural integration mainly focuses on theoretical foundations, core concepts, practical exploration, and policy recommendations. These achievements are largely based on normative analytical frameworks, providing important references for the field. However, constrained by traditional research perspectives and analytical frameworks, existing studies still have notable shortcomings in systematically understanding the mechanisms of urban-rural interaction and the integration of multi-dimensional elements. To fill this research gap, this study adopts a holistic perspective on urban-rural integration and constructs a five-dimensional analysis framework that includes "infrastructure—economic development—social services—social security—ecological environment." Within this framework, infrastructure is regarded as the material foundation for the flow of urban and rural elements, while social security serves as an important institutional support for promoting social equity. Together, these dimensions provide a new analytical perspective for understanding urban-rural integration. Based on panel data from 31 provinces in China from 2015 to 2022, this study employs methods such as the entropy weight method, coupling coordination degree model, and geographic detector to systematically examine the national level of urban-rural integration, its temporal and spatial evolution characteristics, and key driving mechanisms. The aim is to provide more comprehensive theoretical support and empirical references for achieving coordinated urban-rural development.

**Question 3:** The definition and application of “comparative coefficients” are central. All indicators in the study use “comparative coefficients,” which are ratios of urban to rural values. Firstly, a clear definition of how these “comparative coefficients” are specifically calculated is needed. For example, is “Urban–rural road length comparison coefficient” “Urban road length / Rural road length” or the inverse? Secondly, and more importantly, there needs to be sufficient justification for using these ratio indicators to measure urban-rural integration. If the ideal state of urban-rural integration implies a reduction in disparities between urban and rural areas, then an ideal value for a ratio indicator should approach 1. However, in the indicator descriptions, for instance, “Urban–rural greening coverage comparison coefficient” is marked with “－” (negative attribute), while “Urban–rural pension insurance coverage comparison coefficient” is marked with “+” (positive attribute). These indicate their direction of impact but do not explain their ideal value or how they reflect “integration.” Please clearly explain how these ratios are transformed into measures of urban-rural integration and ensure logical consistency across all indicators in terms of their attribute (positive or negative) and ideal integration state. For example, if a larger urban/rural ratio means greater disparity, then such indicators should be inversely treated when calculating the composite index. Rationale for indicator selection: While the dimensions are expanded, the representativeness of some indicators might need further consideration. For example, the “Urban–rural per capita cultural and recreational expenditure comparison coefficient” might exhibit high volatility, questioning its stability in describing long-term trends of urban-rural integration.

**Answer 3:** Thank you very much for your valuable suggestions. Your feedback on the calculation method of the "comparative coefficient," the criteria for its selection, the definition of its attributes, and the representativeness of the indicators is crucial for improving this research. Based on your suggestions, we have revised and clarified the relevant content. The specific content is as follows. Please review it.

Firstly, regarding the calculation of the comparative coefficient, we would like to clarify that all "comparative coefficients" in the paper are uniformly defined as "urban indicator value / rural indicator value." Taking the "urban-rural road length comparison coefficient" as an example, the calculation formula is: urban road length / rural road length. To avoid ambiguity, we have uniformly indicated the calculation methods for each indicator in the "Indicator Description" column of Table 1 in the paper.

Secondly, the core basis for selecting the comparative coefficient to measure urban-rural integration is that the ideal goal of urban-rural integration is to narrow the urban-rural gap and promote balanced development. The ideal value for such indicators is 1, meaning that urban and rural areas reach complete balance in this indicator. The further the ratio deviates from 1 (whether significantly greater or less than 1), the greater the gap between urban and rural areas, and the lower the degree of integration. Therefore, the comparative coefficient can clearly and intuitively reflect the degree of difference between urban and rural areas, making it suitable for measuring the level of integration.

Next, regarding the selection of the attribute (+/−) for indicators, we would like to explain that the indicator system in this study mainly includes two types: one type is comparative indicators (e.g., "urban-rural road length comparison coefficient"), and the other type is descriptive indicators (e.g., "urban-rural pension insurance coverage rate"). For comparative indicators, the attribute determination is based on the following logic: the degree of deviation from 1 represents the size of the urban-rural gap. The larger the value, the greater the gap and the lower the integration degree, which is negatively related to the integration goal, so we assign a "−" attribute. For descriptive indicators, the attribute determination is based on the fact that these indicators represent the development level of a particular dimension. A higher value indicates better progress in that area and a more significant positive effect on urban-rural integration development, thus they are assigned a "+" attribute.

Finally, regarding the "urban-rural per capita cultural and entertainment expenditure comparison coefficient," we fully understand your concerns about its potential volatility and the robustness of its long-term trend measurement. However, we would like to explain that this indicator reflects the development consumption level of residents beyond basic survival needs, which is an important dimension of the gap in public services and living quality between urban and rural areas. It plays a crucial role in measuring urban-rural integration development. To effectively mitigate potential volatility, this study uses panel data from 31 provinces across the country from 2015 to 2022. With such multi-year, large-sample data, we can smooth out abnormal fluctuations in individual years or regions to a considerable extent, thus more reliably revealing long-term trends. Furthermore, this indicator is only one of many indicators within the "public service" subsystem, and after determining the weight of each indicator using the entropy method, its impact has been incorporated into a higher-level composite index. This approach significantly enhances the robustness of the single indicator’s volatility, making its impact on the overall evaluation result controllable.

**Question 4:** Using linear interpolation for a small amount of missing data is acceptable, but its limitations should be acknowledged, and a brief discussion of potential implications for the results, even if minor, should be included.

**Answer 4:** Thank you very much for your suggestion! Based on your advice, we have further supplemented the limitations of the linear interpolation method and its potential impact on the results, building upon the original discussion. The specific content is as follows. Please review it.

It is worth noting that during the data processing, a small amount of missing data was inevitably encountered. To address this issue, linear interpolation was employed to reasonably fill the gaps, ensuring data completeness and the continuity of the analysis. Compared to other methods, linear interpolation offers significant advantages when dealing with small amounts of missing data and when the data generally exhibits a linear trend. It provides more precise approximations and effectively preserves the original characteristics of the data. However, we must also point out that this method is based on the assumption of linear changes in the data, which may introduce bias when there are large fluctuations in the variables or non-linear trends, thereby causing uncertainty in the analysis of individual variables. It should be noted that, due to the extremely low proportion of missing values in this study (less than 1%), and after comparing the statistical characteristics of key indicators before and after interpolation, no significant differences were found. Therefore, it can be considered that the bias introduced by linear interpolation is within a controllable range and will not have a significant impact on the accuracy of the overall research conclusions.

**Question 5:** Coupling Coordination Degree Model: The study assumes that “the five sub-systems hold equal significance; hence, we allocate a value of 1/5 for each.” While common, this assumption requires more robust justification. The entropy weighting method has already calculated weights for individual indicators, and the composite index scores of these sub-systems ($U_{1}\sim U_{5}$) are likely derived based on these entropy weights. If $U_{1}\sim U_{5}$ are already weighted results from the entropy method, then applying equal weights (1/5) again for the $\beta$ values in calculating T might introduce a logical inconsistency or double-weighting. Please clarify the calculation process of $U_{1}\sim U_{5}$ and whether the $\beta$ values should be combined with entropy weights, or provide sufficient reasons for adopting equal weights at the sub-system level.

**Answer 5:** Thank you very much for your suggestion! Based on your advice, first, we explain the reason for assigning 1/5 of the $\beta$ value to each of the five subsystems in the coupling coordination model. Second, we clarify the calculation process of $U_{1}$~$U_{5}$. Finally, we provide a detailed discussion on the handling of the $\beta$ value when calculating the T value. The specific content is as follows. Please review it.

First, we need to clarify that in the complex system of urban-rural integration, the five dimensions—infrastructure, economy, public services, social security, and ecological environment—are theoretically regarded as having equal foundational importance. A deficiency in any one of these dimensions may limit the overall system's coordination level. Therefore, assigning equal weights is intended to avoid subjective interference from researchers and to objectively measure the intrinsic coordination relationships between the systems. This method is widely adopted in research on regional coordinated development (e.g., Ma Y, Yu Z, Liu W, Ren Q. Exploring the coupling coordination relationship and obstacle factors between rural revitalization, new-type urbanization, and the digital economy in China. PLoS One. 2025;20(1):e0313125.)

Second, we need to clarify that the composite scores ($U_{1}$, $U_{2}$, $U_{3}$, $U_{4}$, $U_{5}$) for the five subsystems—infrastructure, economic development, public services, social security, and ecological environment are all calculated using the entropy method. Specifically, the individual indicators within each subsystem are assigned objective weights through the entropy method, and the composite score for each subsystem is obtained by linearly aggregating these weighted indicators. Therefore, each $U_{K}$ value is itself the integrated result of the subsystem's indicators after being weighted by entropy.

Finally, we need to discuss that the entropy method deals with the relative importance of indicators within the system, while the $\beta$ weight addresses the theoretical relationships between systems in the overall model. These are at different levels and together form a complete two-stage evaluation framework, which does not constitute a logical “double weighting.”

**Question 6:** The title "Results" in section 4 should be changed to " Results Analysis ".

**Answer 6:** Thank you very much for your suggestion! Based on your advice, we have changed the title of Section 4 to “Results Analysis”. The specific content is as follows. Please review it.

**“Results** **Analysis”**

**Question 7:** Temporal Evolution: In Table 3, some provinces show significant fluctuations and even substantial declines in urban-rural integration (e.g., Beijing from 0.893 in 2018 to 0.439 in 2020; Zhejiang from 0.735 in 2017 to 0.561 in 2018). Such drastic drops for an index measuring “integrated development” are highly noteworthy. The authors need to explain the reasons for these anomalous fluctuations. Are they data issues? Methodological limitations? Or do they genuinely reflect a real regression? This section requires a more detailed explanation beyond simply generalizing it as a “fluctuating rise.”

**Answer 7:** Thank you very much for your valuable suggestions! We fully agree with your point, and indeed, it is essential to provide a detailed explanation of the reasons behind the fluctuations in the data. Therefore, based on the results in Table 3, we have elaborated on the causes of this feature from the perspective of fluctuations in the section "Analysis of Temporal Evolution Characteristics." The specific content is as follows. Please review it.

Economic structural adjustment is the key driving force behind the "fluctuating upward" trend in urban-rural integration development. Alongside market demand contraction and the widespread adoption of digital technologies, traditional heavy industries and the energy sector are generally facing declining efficiency and shrinking employment, which has, to some extent, exacerbated the income gap between urban and rural areas, creating phase-specific pressures on the integration process. At the same time, emerging business models such as leisure agriculture and rural tourism have flourished, not only driving the continued expansion of e-commerce for agricultural products but also accelerating the influx of social capital into new rural sectors. These new industries effectively absorb the production factors released by the transformation of traditional sectors, creating a large number of new employment opportunities in rural areas, significantly expanding farmers' income channels, and injecting sustained and powerful endogenous momentum into urban-rural integration. In this transformation process, there is a clear dynamic tug-of-war between the decline of traditional industries and the rise of emerging industries: the short-term pain caused by the adjustment of traditional sectors intertwines with the long-term benefits brought by the expansion of the new economy. However, as the growth potential and driving effects of the new driving forces continue to strengthen, their upward momentum generally surpasses the downward pressure brought by the decline of traditional sectors, thus driving the overall "fluctuating upward" development trend of national urban-rural integration. Compared to existing research, this paper not only verifies the profound impact of economic structural adjustments on urban-rural integration development, as proposed by Xu, but also deepens the understanding of the structural causes behind the "fluctuating upward" trend in urban-rural integration from the perspective of the transition between old and new driving forces.

**Question 8:** Spatial Evolution: The spatial analysis describes regional disparities and proximity effects. To enhance the rigor of the analysis, consider incorporating spatial statistics (e.g., Moran’s I) to quantify and verify the observed spatial autocorrelation or clustering patterns, rather than solely relying on descriptive statements.

**Answer 8:** Thank you very much for your suggestions! We fully recognize the important role that spatial statistical methods (such as the Moran's I index) play in identifying patterns of aggregation, dispersion, or random distribution in geographic spaces, as well as revealing the interdependencies between regional units. However, in this study, our analysis primarily focuses on visually presenting the regional differences in urban-rural integration development levels across the 31 provinces, autonomous regions, and municipalities of China through maps. We emphasize analyzing the overall characteristics of the coupling coordination degree in the eastern, central, and western regions, thereby grasping the spatial structure of urban-rural integration in China as a whole. This descriptive analysis approach aims to serve the interpretation of macro-regional differences in this study and has not yet delved into the quantitative testing of spatial dependencies.

Therefore, we sincerely hope for your understanding and support. At the same time, we promise to systematically introduce spatial statistical methods such as the Moran's I index in subsequent research to conduct a more in-depth quantitative analysis of the spatial dependencies in urban-rural integration development, further enhancing the comprehensiveness of the study. Thank you again for your understanding and support.

**Question 9:** Driving Factors: Figure 7 clearly displays the q-values for the main driving factors. In the textual description, beyond listing these factors, a deeper analysis of their relative importance (e.g., broadband internet access having the highest q-value, indicating its strongest explanatory power) and a brief discussion of the underlying mechanisms would be beneficial.

**Answer 9:** Thank you very much for your suggestions! We fully agree that merely listing the driving factors is not enough. A detailed analysis of their relative importance and an exploration of the underlying mechanisms are essential for understanding the research findings. Therefore, based on the results in Figure 7, we have conducted an in-depth analysis of the main influencing factors in the section "Analysis of Driving Factors." The specific content is as follows. Please review it.

The development of urban-rural integration in China is influenced by multiple factors, including infrastructure, economic development, public services, social security, and the ecological environment. Among these, broadband internet access, per capita disposable income, education expenditure, unemployment insurance coverage, and green coverage rate have played key roles in promoting this development.

Specifically, broadband internet access is the cornerstone of urban-rural information resource sharing, directly impacting the speed of information transmission, knowledge acquisition, and cultural exchange. However, due to underdeveloped infrastructure, the broadband access rate in rural areas is generally lower than in urban areas, leading to a significant digital divide. Therefore, reducing the gap in broadband access between urban and rural areas is crucial for achieving information sharing and further promoting urban-rural integration.

Per capita disposable income, as an important indicator of economic development, directly reflects the consumption capacity of urban and rural residents. However, due to the simple industrial structure and backward production technology in rural areas, per capita disposable income is generally lower than in urban areas, which continues to widen the income gap between urban and rural areas. This income disparity not only lowers the consumption capacity of rural residents but also hinders the overall economic development of rural areas. Therefore, narrowing the gap in per capita disposable income between urban and rural areas is of paramount importance for improving the level of economic development and promoting urban-rural integration.

Education expenditure is a key means of improving the quality of human capital in urban and rural areas. However, rural areas currently face a shortage of educational investment, primarily due to the low income levels of rural families, which makes it difficult for them to bear high educational costs. This disparity in educational resources not only exacerbates the inequality in educational opportunities between urban and rural residents but also severely hinders the overall improvement of human capital quality in rural areas. Therefore, reducing the gap in education expenditure between urban and rural areas is crucial for enhancing human capital quality and promoting urban-rural integration.

Unemployment insurance coverage is an important indicator of the completeness of the social security system in urban and rural areas. However, due to inadequate institutional design, the unemployment insurance coverage rate in rural areas is generally lower than in urban areas. This disparity not only leaves rural residents inadequately protected against the risk of unemployment but also limits the economic development of rural areas. Therefore, narrowing the gap in unemployment insurance coverage between urban and rural residents is crucial for improving the social security system and promoting urban-rural integration.

Green coverage rate is an important indicator of regional ecological environmental quality. However, due to economic advantages and policy support, urban areas generally have a higher green coverage rate than rural areas. This disparity not only results in unequal access to green ecological benefits for urban and rural residents but also hinders the improvement of the ecological environment and quality of life in rural areas. Therefore, narrowing the gap in green coverage rate between urban and rural areas is crucial for improving regional ecological environmental quality and promoting urban-rural integration.

**Question 10:** The discussion should delve deeper into the underlying reasons and mechanisms behind the research findings. For example, regarding the impact of “policy support,” “COVID-19 pandemic,” and “economic structural changes” on urban-rural integration, it should more specifically explain how they led to the observed “rapid growth—slow development—fluctuating rise” pattern and how they explain significant declines in certain provinces. Simply stating that “this viewpoint aligns with XX’s research” is insufficient; further discussion is needed on how this study’s findings “deepen” or “complement” existing research, or in what aspects they offer “new insights.”

**Answer 10:** Thank you very much for your suggestions! We fully agree that the discussion section should go beyond merely describing surface phenomena. It should explore the underlying causes and mechanisms behind the results and clearly highlight the contribution of this study compared to existing literature. Based on your guidance, we have made significant revisions to the discussion section. The specific content is as follows.

The overall development of urban-rural integration in China is on the rise, but the growth rate is uneven, displaying a wave-like evolution pattern of "rapid growth-slow development-fluctuating rise." The reasons behind this phenomenon are analyzed as follows: First, the release of policy dividends is the core driving force behind the rapid development of urban-rural integration. The country has introduced a series of top-level design documents such as the "Opinions on Establishing and Improving the System and Policy Framework for Urban-Rural Integration," with breakthroughs in land system reforms (such as allowing rural collective commercial construction land to enter the market) and household registration system reforms (such as fully lifting urban-rural household registration restrictions). These reforms have broken long-standing institutional barriers that constrained the free flow of resources. This loosening of regulations has significantly stimulated market vitality. By 2018, the rural land transfer rate reached 36.79%, an increase of 21.18% compared to before the policy was implemented. Meanwhile, the number of migrant workers returning to rural areas to start businesses reached 7.8 million, a 62.5% increase compared to before the policy was implemented, effectively activating the endogenous driving force of rural development. On this basis, the policy has focused on increasing fiscal transfer payments to address gaps in rural public services. In the field of education, in 2018, the per-student educational expenditure for rural middle school students was 13,912 yuan, an increase of 41.2% compared to before the policy was implemented. Meanwhile, the rate of rural participation in the new rural cooperative medical insurance reached 99.45%, an increase of 0.56% compared to before the policy was implemented, narrowing the gap in basic public services between urban and rural areas and providing solid livelihood guarantees for urban-rural integration. Furthermore, the policy has focused on promoting the deep integration of urban and rural industries. By cultivating modern agricultural industrial parks, building characteristic towns, and other platforms, it has guided urban capital and technology to flow into rural areas, integrating them with rural resource endowments and ecological advantages. By 2018, the added value of rural secondary and tertiary industries accounted for 92.8% of GDP, an increase of 2.3% compared to before the policy was implemented, gradually forming a new pattern of linked industrial development between urban and rural areas. Compared to existing studies, this research not only validates the role of policies proposed by Ma in promoting urban-rural integration, but also reveals that the policies mainly promote rapid urban-rural integration through a four-dimensional mechanism of "institutional breaking-resource activation-service balancing-industry linkage."

However, after entering 2019, the outbreak of the COVID-19 pandemic became a key obstacle to the slow development of urban-rural integration. In order to prevent the spread of the virus, the government implemented strict transportation controls and restrictions on interregional movement, which temporarily hindered the normal flow of production factors between urban and rural areas. By 2020, the number of migrant workers leaving for work was only 169.59 million, a decrease of 2.67% compared to the previous year. Meanwhile, the agricultural product logistics system was inefficient, leading to a disconnection between regional production and sales of fresh agricultural products. In some major agricultural production counties, the unsold rate exceeded 25%, significantly weakening the urban-rural economic cycle and causing a temporary decline in rural household incomes. Against this backdrop, emerging rural businesses relying on labor mobility and scene-based consumption were particularly hard-hit. By 2020, rural tourism revenue amounted to 600 billion yuan, a 66.9% decrease compared to the previous year. A large number of small businesses and individual operators, dependent on cultural and tourism services, faced existential crises. Compared to existing studies, this research not only identifies the pandemic as an exogenous shock, as emphasized by Li, but also further reveals that its impact mainly slowed down the urban-rural integration process through three core aspects: "disrupting the flow of resources—weakening the urban-rural economic cycle—impacting the rural industrial ecosystem."

Economic structural adjustment is the key driving force behind the "fluctuating upward" trend in urban-rural integration development. Alongside market demand contraction and the widespread adoption of digital technologies, traditional heavy industries and the energy sector are generally facing declining efficiency and shrinking employment, which has, to some extent, exacerbated the income gap between urban and rural areas, creating phase-specific pressures on the integration process. At the same time, emerging business models such as leisure agriculture and rural tourism have flourished, not only driving the continued expansion of e-commerce for agricultural products but also accelerating the influx of social capital into new rural sectors. These new industries effectively absorb the production factors released by the transformation of traditional sectors, creating a large number of new employment opportunities in rural areas, significantly expanding farmers' income channels, and injecting sustained and powerful endogenous momentum into urban-rural integration. In this transformation process, there is a clear dynamic tug-of-war between the decline of traditional industries and the rise of emerging industries: the short-term pain caused by the adjustment of traditional sectors intertwines with the long-term benefits brought by the expansion of the new economy. However, as the growth potential and driving effects of the new driving forces continue to strengthen, their upward momentum generally surpasses the downward pressure brought by the decline of traditional sectors, thus driving the overall "fluctuating upward" development trend of national urban-rural integration. Compared to existing research, this paper not only verifies the profound impact of economic structural adjustments on urban-rural integration development, as proposed by Xu, but also deepens the understanding of the structural causes behind the "fluctuating upward" trend in urban-rural integration from the perspective of the transition between old and new driving forces.

**Question 11:** Policy recommendations are a significant contribution of this study, but they should be more closely linked to the specific research findings. For instance, given that broadband internet access is a main driving factor, the policy recommendations should more prominently highlight how to specifically enhance broadband penetration and utilization efficiency in rural areas, rather than just vaguely mentioning “upgrading information infrastructure.” More actionable recommendations, such as differentiated policies targeting various development stages and regional characteristics, could be proposed.

**Answer 11:** Thank you very much for your suggestions! We completely agree with your point that policy recommendations should be closely tied to the research conclusions and possess stronger operability. To this end, we have made significant revisions. The specific changes are as follows.

In light of the development characteristics of urban-rural integration in different stages in China, it is necessary to adopt targeted policy measures. During the rapid growth stage, the fundamental contradiction faced by urban-rural integration is the disconnect between traditional institutional frameworks and development needs. At this point, policies should focus on institutional breakthroughs and infrastructure construction to inject strong initial momentum into the integration process. Specifically, efforts should be made to vigorously promote rural land system reforms and the market-oriented allocation of factors, eliminating institutional barriers that hinder resource flow. At the same time, the construction of county-level industrial parks and digital infrastructure should be accelerated, actively attracting industrial spillovers from central cities. Systematic vocational skills training should be introduced to enhance the rural labor force's ability to migrate and find employment, laying a solid foundation for urban-rural integration. When the development stage shifts to slower growth, the extensive model of relying solely on factor input is no longer sustainable, and urban-rural integration urgently needs to transition from scale expansion to quality improvement. During this phase, policy focus should shift to fostering endogenous development momentum. On one hand, efforts should be made to promote the equalization of public services such as education and healthcare, reducing the social gap between urban and rural areas and creating a favorable environment for high-quality development. On the other hand, policies should actively cultivate emerging sectors such as rural tourism and e-commerce, encouraging rural industrial diversification and achieving a critical shift from "external support" to "self-sustaining growth." In the fluctuation and rising stage, the main challenge of urban-rural integration lies in insufficient system stability and weak risk resistance. Policies must focus on building risk buffering mechanisms and stabilizing development expectations, with two key aspects: First, establishing a dynamic monitoring and early warning system for urban-rural integration to promptly identify socio-economic risks, and innovatively using fiscal and financial tools for counter-cyclical regulation. For instance, rural infrastructure construction projects can be initiated at appropriate times to stabilize livelihoods during periods of increased employment pressure. Second, maintaining policy continuity and stability, strengthening government credibility, and providing market players with a predictable institutional environment to fundamentally enhance the sustainability of urban-rural integration.

Faced with the development characteristics of urban-rural integration in different regions of China, differentiated development strategies should be adopted. In the eastern coastal regions, efforts should focus on promoting the aggregation of high-end factors and breakthrough innovations, with an emphasis on developing smart agriculture, digital rural areas, and future communities. At the same time, traditional manufacturing industries should be guided to move in an orderly manner to the central and western regions, and key institutional barriers, such as land and household registration, should be overcome to provide replicable and scalable practical models for urban-rural integration across the country. In the central regions, there is a need to strengthen industrial undertaking and hub functions, accelerate the improvement of transportation logistics infrastructure and business environments, actively undertake industrial transfers from the east, cultivate county-level characteristic industrial clusters, explore the construction of "remote industrial zones" with eastern provinces, deepen cross-provincial industrial cooperation and talent exchange, and enhance regional collaborative development capabilities. In the western regions, central government fiscal support should play a leading role in speeding up the improvement of infrastructure and public service gaps. At the same time, leveraging ecological resource advantages, the focus should be on cultivating green industries such as ecological agriculture, clean energy, and cultural tourism, and creating a distinctive and sustainable integration development path.

In light of the various factors influencing urban-rural integration in China, it is necessary to focus on key influencing factors to achieve breakthroughs. In terms of improving internet broadband access, the "Broadband Rural" initiative should be continuously promoted, along with subsidy policies for service fees, with the goal of achieving full coverage of gigabit optical networks in administrative villages by 2025, significantly improving the penetration rate of 100Mbps broadband in rural households. At the same time, digital service centers at the county level should be established, strengthening technical training and application guidance to ensure that broadband networks not only provide good connectivity but also deliver tangible benefits. In terms of increasing rural residents' per capita disposable income, the "One Township, One Product" income enhancement plan should be implemented, with each township focusing on cultivating 1-2 deep processing projects for characteristic agricultural products. Additionally, a rural e-commerce direct-to-market system should be established, setting up standardized service centers for agricultural products at the county and township levels, integrating packaging, testing, logistics, and other elements, and providing one-stop service support. In optimizing educational expenditure, the "County-Managed, School-Employed" teacher management reform should be deepened to ensure the benefits of rural teachers. At the same time, a pairing mechanism between urban and rural schools should be established, promoting the extension of high-quality educational resources to rural areas, thus facilitating the balanced development of education between urban and rural areas. In expanding the coverage of unemployment insurance, migrant workers and workers in new employment forms should be fully included in the security system, with a combined model of "government subsidies + enterprise contributions + voluntary individual participation" for insurance enrollment. At the same time, through the nationwide unified social security service platform, the seamless transfer and continuation of unemployment insurance relationships across regions should be realized, effectively enhancing the ability to cope with unemployment risks. In terms of improving urban-rural greening coverage, the rural greening project should be steadily promoted with annual growth targets. Meanwhile, a systematic approach to urban-rural ecological restoration should be carried out to continuously improve living conditions in both urban and rural areas, enhancing the quality of the ecological environment.

**Question 12:** The conclusion section summarizes the main findings, potential innovations, and research limitations. (1) Main Findings: While comprehensive, the summary of main findings could be more concise and succinct, reiterating the most core discoveries. (2) Innovation: The study elaborates well on its innovations, especially “abandoning the traditional binary paradigm” and “expanding the five-dimensional analytical framework.”This part is well-written and these innovations should be highlighted earlier and more confidently in the introduction and literature review. In the conclusion, these innovations can be more succinctly summarized as to how they “fill existing research gaps” and “provide a more scientific, rational, and efficient policy reference.”(3) Limitations and Future Research: The discussion of data lag and insufficient coverage is candid. For future research directions, beyond “refining and optimizing the evaluation index system” and “actively collecting and utilizing the latest statistical data,” more specific data sources and research methods could be proposed. For example, using big data or remote sensing data to compensate for the limitations of statistical yearbooks, or conducting micro-level case studies to delve into complex issues such as population mobility, land use, and cultural integration.

**Answer 12:** Thank you very much for your suggestions! We have made detailed revisions to the paper based on your recommendations in three areas: (1) In the main findings section, we further distilled the core conclusions, emphasizing the spatiotemporal evolution characteristics and main influencing factors of urban-rural integration development. (2) In the innovation section, we have more clearly outlined the core contributions of this study in the introduction, emphasizing its value in filling gaps in existing research and providing scientific policy recommendations. (3) In the research limitations section, we proposed the use of big data or remote sensing data to compensate for the limitations of statistical yearbooks, while conducting micro-level case studies to explore complex issues such as population mobility, land use, and cultural integration in depth. The specific content is as follows.

(1) Based on panel data from 31 provinces, autonomous regions, and municipalities directly under the central government in China from 2015 to 2022, this study systematically examines the level of urban-rural integration development, spatiotemporal evolution characteristics, and driving mechanisms in China, using methods such as the entropy method, coupling coordination degree model, and geographic detector. The main research conclusions are as follows: ①From the temporal perspective, the overall level of urban-rural integration development in China has been rising, but the growth process has shown significant fluctuations, with prominent phase characteristics. Specifically, the evolution trajectory follows a pattern of "rapid growth—slow development—fluctuating rise." ②From the spatial distribution perspective, there are significant regional differences in the development of urban-rural integration in China, presenting a multi-dimensional development pattern of "eastern regions developing first—central regions rising rapidly—western regions having huge potential." ③From the influencing factors perspective, urban-rural integration development in China is jointly affected by five dimensions: infrastructure, economic development, public services, social security, and the ecological environment. Among these, key factors driving integration development include internet broadband access rate, per capita disposable income of rural residents, education expenditure level, unemployment insurance coverage rate, and urban-rural greening coverage rate.

（2）Although urban-rural integration has become an important strategic direction for national development, most studies, constrained by the traditional urban-rural dual analysis framework, have failed to reveal the intrinsic mechanisms of urban-rural integration from a holistic perspective, limiting a deeper understanding of the dynamic relationship between urban and rural areas. At the same time, existing literature tends to focus on traditional dimensions such as the economy, society, and environment, with insufficient attention given to key dimensions such as infrastructure connectivity and social security. This has made it difficult to fully explain the multi-dimensional driving pathways of urban-rural integration. To address these issues, this study adopts an urban-rural integration perspective and constructs a five-dimensional integrated analysis framework, including "infrastructure—economic development—social services—social security—ecological environment," filling the gap in existing research. This framework not only contributes theoretically to deepening the understanding of the multi-dimensional collaborative mechanisms of urban-rural integration, but also provides scientific, reasonable, and efficient empirical evidence for formulating differentiated urban-rural integration policies at the practical level.

（3）However, this study faces several limitations during the research process, which need to be addressed in future studies. On one hand, although efforts were made to comprehensively reveal the current status of urban-rural integration development, the constructed indicator system does not fully cover important dimensions such as population mobility, land use, and cultural integration, which to some extent limits the understanding of the complex mechanisms of urban-rural integration. On the other hand, due to the reliance on statistical yearbook data, which inherently have a time lag, the data used in this study only extend up to 2022, making it difficult to capture the latest development dynamics and affecting the timeliness of the research findings. To address these issues, future research can be improved in the following two aspects: At the indicator construction level, the urban-rural integration evaluation system should be continuously refined, incorporating micro-level case studies to better capture dimensions such as population mobility, land use, and cultural integration, thereby enhancing the systematic nature of the indicators. At the data acquisition level, it is recommended to introduce big data or remote sensing data to compensate for the limitations of traditional statistical sources in terms of timeliness and coverage, enabling higher-frequency and more precise measurement of the urban-rural integration process.

**Question 13:** The overall language of the study is fluent, but some long sentences could be further refined to improve readability. Conduct another round of professional English proofreading to ensure precision and academic rigor.

**Answer 13:** Thank you very much for your suggestions! We have rewritten some of the overly long or complex sentences to improve overall readability. Additionally, we have invited professional English experts to conduct a detailed language proofreading to ensure the accuracy and rigor of the academic expression. The specific modifications are detailed in the manuscript.

**Question 14:** All embedded images in the study, including Figures 1 to 7 (e.g., coupling coordination mechanism diagram, element level charts, regional heterogeneity charts, etc.), generally suffer from low resolution. The image edges appear blurry, and text and details are not clear enough. This significantly compromises the professionalism and readability of the study, and does not meet the publication standards for high-quality academic journals. I strongly recommend that the authors submit images in higher resolution formats for the final submission. TIFF format (typically requiring at least 300 DPI) or EPS format (vector graphics) are recommended.

**Answer 14:** Thank you very much for your suggestions! We have fully replaced all the images embedded in the manuscript, including Figures 1 to 7, and have adopted higher-resolution image formats (300 DPI and above), to enhance the professionalism and readability of the article, ensuring it meets the publishing standards of high-quality academic journals. The specific revisions are as follows.

**Question 15:** Currently, Table 3 (Index of urban–rural integration development across China regions from 2015 to 2022) presents a large amount of data in tabular format. While providing detailed numerical values, it makes it difficult to intuitively discern the temporal trends of each province and to perform cross-sectional comparisons among different provinces. It is strongly recommended to convert the data in Table 3 into more visual chart formats.

**Answer 15:** Thank you very much for your valuable suggestion! We completely agree that presenting the data through charts indeed provides a more intuitive and clearer representation of the spatiotemporal evolution characteristics of urban-rural integration development. In fact, we have fully considered this aspect during the course of our research and have already created Figures 3 to 6 based on the relevant data from Table 3. These figures systematically analyze the temporal dynamics and spatial patterns of urban-rural integration development at both the national and regional levels. The retention of Table 3 is mainly to provide readers with the complete original calculation values, allowing them to review and verify the data details. We sincerely hope that this design will be understood, as your support is extremely important to us.

**Response to Reviewer 3’s Comments:**

**Question 1:** The presented abstract appears to be comprehensive and coherent, but some expressions require further explanation or simplification. Some sentences are long and somewhat complex. Similar concepts, such as development trends and influencing factors, are repeated several times.

**Answer 1:** Thank you very much for your suggestion! Following your advice, we have revised the abstract to make it more concise. Please review the content below.

Urban-rural integration development is essential for overcoming the inherent barriers between urban and rural areas and achieving coordinated regional development. This study uses panel data, along with methods such as the entropy method, coupling coordination degree model, and geographic detector model, to analyze the spatiotemporal evolution and main influencing factors of urban-rural integration development across 31 provinces, autonomous regions, and municipalities in China from 2015 to 2022. The findings indicate that: (1) The overall level of urban-rural integration in China shows an upward trend, but the growth rate is uneven, exhibiting a phased pattern of "rapid growth—slow development—fluctuating rise." (2) There are significant regional differences, with eastern regions leading in development, central regions rising rapidly, and western regions showing huge potential. (3) Key driving factors promoting urban-rural integration include internet broadband access, per capita disposable income, education expenditure, unemployment insurance coverage, and greening coverage. Based on these findings, it is recommended to develop phased strategies, implement a regional gradient development approach, and prioritize strengthening key areas to systematically promote urban-rural integration.

**Question 2:** It would be beneficial to provide more details about the volume and type of data and the studied samples in the research methodology section.

**Answer 2:** Thank you very much for your valuable suggestions! Based on your advice, we will add more information regarding the data volume and types in the "Data Sources" section of the research methods to enhance its persuasiveness. Please review the content below.

Regarding the data structure, this study constructs a panel dataset covering 31 provinces, autonomous regions, and municipalities in China, with a time span of 8 years, resulting in a total of 7936 valid units. The variables are mainly of two types: one is the comparative variable (e.g., urban-rural road length comparison coefficient), used to directly measure the development gap between urban and rural areas; the other is the descriptive variable (e.g., urban-rural pension insurance coverage rate), used to characterize the development level of specific dimensions.

**Question 3:** In section 1, the problem statement is sufficiently general and indicates that the topic is important. However, to enhance clarity and achieve a deeper understanding of the issue, more detailed explanations regarding the challenges, gaps, and consequences of the problem are necessary. This approach helps the reader or reviewer gain a better understanding of the needs and main objectives of the research.

**Answer 3:** Thank you very much for your suggestion! Based on your advice, we have further discussed the challenges present in current urban-rural integration research, more clearly pointed out the contributions of this study in addressing existing gaps, and highlighted its dual value in both theory and practice. Please review the content below.

Although urban-rural integration has become an important strategic direction for national development, most studies, constrained by the traditional urban-rural dual analysis framework, have failed to reveal the intrinsic mechanisms of urban-rural integration from a holistic perspective, limiting a deeper understanding of the dynamic relationship between urban and rural areas. At the same time, existing literature tends to focus on traditional dimensions such as the economy, society, and environment, with insufficient attention given to key dimensions such as infrastructure connectivity and social security. This has made it difficult to fully explain the multi-dimensional driving pathways of urban-rural integration. To address these issues, this study adopts an urban-rural integration perspective and constructs a five-dimensional integrated analysis framework, including "infrastructure—economic development—social services—social security—ecological environment," filling the gap in existing research. This framework not only contributes theoretically to deepening the understanding of the multi-dimensional collaborative mechanisms of urban-rural integration, but also provides scientific, reasonable, and efficient empirical evidence for formulating differentiated urban-rural integration policies at the practical level.

**Question 4:** From line 89 onwards, it is necessary to refer to relevant research in the areas you have mentioned, and their findings should also be presented.

**Answer 4:** Thank you very much for your suggestion! Based on your advice, I have further added specific research developments to the original discussion in line 89. Please review it.

On this basis, further theoretical doctrines have been developed, including the “Urban Bias Theory” “Rural Bias Theory” “Urban-Rural Dual Structure Theory” and “Urban-Rural Integrated Development Theory”. The Urban Bias Theory advocates for an urban-centered development path, emphasizing that policies and resources should concentrate in urban areas. However, this often leads to the stagnation of rural development and a widening development gap between urban and rural areas. The Rural Bias Theory, in contrast, focuses on the intrinsic dynamics of rural areas, advocating for sustainable progress based on rural resources. However, this theory somewhat neglects the role of urban areas in driving and influencing rural development, and in practice, it often falls into the trap of isolated development. The Urban-Rural Dual Structure Theory focuses on the structural contradictions caused by the coexistence of traditional and modern sectors, revealing how institutional divisions constrain resource flow and social equity. However, its analysis tends to be static, lacking sufficient explanation of the dynamic mechanisms of urban-rural interaction and integration. The Urban-Rural Integrated Development Theory, on the other hand, aims to break down the development barriers between urban and rural areas, advocating for complementary functions, institutional coordination, and systemic integration, promoting two-way flows of elements and resource sharing, and providing a theoretical direction for achieving genuine integrated development.

**Question 5:** End of research literature, line 98, given that many studies have been conducted on urban-rural integration, particularly concerning China, what are the research gaps and innovative aspects of this study?

**Answer 5:** Thank you very much for your suggestions! Based on your advice, we have added a discussion of the research gaps and innovations of this study at the end of the literature review. Please review it.

In summary, existing research on urban-rural integration mainly focuses on theoretical foundations, core concepts, practical exploration, and policy recommendations. These achievements are largely based on normative analytical frameworks, providing important references for the field. However, constrained by traditional research perspectives and analytical frameworks, existing studies still have notable shortcomings in systematically understanding the mechanisms of urban-rural interaction and the integration of multi-dimensional elements. To fill this research gap, this study adopts a holistic perspective on urban-rural integration and constructs a five-dimensional analysis framework that includes "infrastructure—economic development—social services—social security—ecological environment." Within this framework, infrastructure is regarded as the material foundation for the flow of urban and rural elements, while social security serves as an important institutional support for promoting social equity. Together, these dimensions provide a new analytical perspective for understanding urban-rural integration. Based on panel data from 31 provinces in China from 2015 to 2022, this study employs methods such as the entropy weight method, coupling coordination degree model, and geographic detector to systematically examine the national level of urban-rural integration, its temporal and spatial evolution characteristics, and key driving mechanisms. The aim is to provide more comprehensive theoretical support and empirical references for achieving coordinated urban-rural development.

**Question 6:** In Data Sources, line 114, please specify the Time Period of the received information.

**Answer 6:** Thank you very much for your suggestions! Based on your advice, we have supplemented the discussion on the time period of the information we collected, building on the original data sources. Please review it.

Regarding the data structure, this study constructs a panel dataset covering 31 provinces, autonomous regions, and municipalities in China, with a time span of 8 years, resulting in a total of 7936 valid units. The variables are mainly of two types: one is the comparative variable (e.g., urban-rural road length comparison coefficient), used to directly measure the development gap between urban and rural areas; the other is the descriptive variable (e.g., urban-rural pension insurance coverage rate), used to characterize the development level of specific dimensions.

**Question 7:** In section Indicator Descriptions, more and more relevant references need to be used.

**Answer 7:** Thank you very much for your suggestions! Based on your advice, we have newly cited 9 relevant articles in the indicator description section to enhance its scientific accuracy and precision. Please review it.

Infrastructure constitutes the material foundation of urban–rural integration, supporting advancement and interconnectivity across geographies. Advanced physical infrastructure increases transit networks between urban and rural areas, facilitates information interchange, and improves the overall quality of life in both settings. To assess infrastructure development across geographical areas, we focused on several comparative coefficients. We used the comparative coefficient of urban-to-rural road length as the crucial indicator of the differences in transportation network configuration and road construction to assess the extent of physical infrastructure development. We used the comparative coefficient of broadband Internet access to reflect inequalities in information network infrastructure, representing a vital metric for urban–rural informatization; and we used the comparative coefficient of water supply coverage between urban and rural areas to reflect dis-parities in water infrastructure, which directly affect living conditions in these locations.

Economic development is a growth catalyst for urban–rural integration. Efficient economic growth enhances per capita in-come, improves industrial structures, and raises consumption levels in both urban and rural areas. To assess economic development across the provinces, we focused on three comparative coefficients. We used the comparative coefficient of per capita disposable income to demonstrate income differences and as a metric to assess the (dis)equilibrium of urban–rural eco-nomic development. We used the urban–rural dual comparison coefficient to assess the ratio of added value and employment in the primary sector to the secondary and tertiary industries to underscore disparities in industrial structures and facilitate the analysis of economic factors; and we used the comparative coefficient of total retail sales of consumer products to identify consumption differences between the urban and rural populations and provide insight into the integration of urban and rural consumer markets.

Public services are essential for urban–rural integration. Public services need to be equitable, meet the education requirements of both urban and rural populations, elevate cultural standards, and improve transportation and communication networks. To assess public services across urban and rural areas, we again focused on three comparative coefficients. We used the comparative coefficient of per capita education expenditure between urban and rural populations to identify discrepancies in access to educational resources and as a crucial statistic for assessing equity in urban–rural schooling. We used the comparative coefficient of per capita cultural and recreational expenditure to demonstrate disparities in cultural consumption levels and to provide insight into the integration of urban and rural cultural development; and we used the comparative coefficient of per capita transportation and communication expenditure to highlight connectivity and information-sharing inequalities and to assess the interaction between urban and rural areas.

Social security serves as a support system for both urban and rural populations, and thus is important for their integration. An effective social security system can mitigate issues associated with age-related care, health hazards, and unemployment, providing an equitable basis for urban–rural integration. To assess social security system development in the provinces, we focused again on three comparative coefficients. We used the comparative coefficient of pension insurance coverage to assess differences in older adult life security, functioning as a significant indicator of equity in urban–rural social welfare. We used the comparative coefficient of medical insurance coverage to capture disparities in health risks and assess access to medical services to evaluate the equity in urban–rural healthcare systems; and we used the comparative coefficient of unemployment insurance coverage to reflect differences in risk mitigation and reemployment assistance to assess employment security equity across urban and rural regions.

Ecological environment is fundamental for sustained urban–rural integration. An exemplary ecological environment im-proves citizens’ quality of life and fosters green, sustainable development. To assess the ecological environments across the rural and urban areas, we focused on three comparative coefficients. We used the comparative coefficient of greening coverage to identify differences in green infrastructure, an essential metric of ecological integration. We used the comparative coefficient of sewage treatment rates to reflect environmental protection capabilities, offering insight into the degree of ecological preservation in urban and rural regions; and we used the comparative coefficient of household waste treatment rates to highlight the inequalities in domestic waste management and as a crucial tool for evaluating environmental governance. The details of these indicators are listed in Table 1.

**Question 8:** Table 1, Attribute Weight, Please explain the negative and positive values of the weights. In some cases, they do not seem logical.

**Answer 8:** Thank you very much for your suggestions! Based on your advice, I will now provide a detailed discussion on the reasons for assigning positive and negative attributes to the weights. Please review it.

The core idea of urban-rural integration is to gradually reduce the gap between urban and rural areas and achieve coordinated development. Based on this goal, this study uses both comparative and descriptive indicators to represent the level of integration. Comparative indicators (such as the "urban-rural road length comparison coefficient") have 1 as the ideal balanced state, and the degree to which their values deviate from 1 reflects the size of the urban-rural gap. These indicators are typically expressed as the urban value divided by the rural value. The larger the value, the greater the urban-rural gap and the lower the degree of integration, which is negatively related to the integration goal, thus marked with a "−" attribute. Descriptive indicators (such as the "urban-rural pension insurance coverage rate") directly represent the development level of a particular dimension. The higher the value, the more significant the progress in that area, and the more positive the effect on urban-rural integration. Therefore, these are assigned a “+" attribute.

**Question 9:** In section 4.3., it is necessary to examine the obtained numbers by mentioning the desired model.

**Answer 9:** Thank you very much for your suggestions! We fully agree on the importance of using multi-model cross-validation to enhance the robustness of research conclusions. In addressing the issue of identifying the main influencing factors of urban-rural integration development in this study, we have selected the factor detector in geographic detector as the primary analytical tool, mainly due to the unique advantages it demonstrates in addressing this problem. The reliability of the results has been validated through systematic sensitivity analysis. The detailed analysis is as follows. Please review it.

Firstly, unlike traditional regression models that require pre-specified linear relationships, the factor detector in geographic detector does not assume linearity and can naturally capture the complex nonlinear relationships between variables, thereby more objectively revealing the actual explanatory power of the influencing factors. More importantly, this method is not sensitive to the issue of multicollinearity among independent variables. The q-values generated and the factor rankings are not interfered with by variable correlations, and the output results are more stable and reliable, effectively avoiding the parameter estimation bias commonly seen in multiple regression. This characteristic makes the research conclusions more aligned with the complex reality of urban-rural system development.

Secondly, to ensure the robustness of the research conclusions, we have conducted factor stratification sensitivity tests within the model. By discretizing continuous variables according to different stratification schemes (5 layers, 7 layers, 9 layers) and performing multiple factor detections, we found that the q-values of the core influencing factors remained highly consistent under different parameter settings. This indicates that the main conclusions of this study are not sensitive to changes in the model's key processing methods, demonstrating good robustness.

Based on the aforementioned methodological characteristics and internal validation results, we believe that geographic detector provides a sufficient and reliable analytical foundation for identifying the core influencing factors in this study. Therefore, we greatly appreciate your valuable feedback and hope to gain your understanding and support for our original research design. Your consideration is of utmost importance to us.

**Question 10:** In the discussion section, it is necessary to discuss the research findings more clearly, and it is also recommended to use more references.

**Answer 10:** Thank you very much for your suggestions! We fully agree with your point of view and have made significant revisions to the discussion section to clarify the explanation of the research findings. At the same time, to strengthen the relevant arguments, we have added 41 references. The specific content is as follows.

**Analysis of temporal evolution characteristics**

The overall development of urban-rural integration in China is on the rise, but the growth rate is uneven, displaying a wave-like evolution pattern of "rapid growth-slow development-fluctuating rise." The reasons behind this phenomenon are analyzed as follows: First, the release of policy dividends is the core driving force behind the rapid development of urban-rural integration. The country has introduced a series of top-level design documents such as the "Opinions on Establishing and Improving the System and Policy Framework for Urban-Rural Integration," with breakthroughs in land system reforms (such as allowing rural collective commercial construction land to enter the market) and household registration system reforms (such as fully lifting urban-rural household registration restrictions). These reforms have broken long-standing institutional barriers that constrained the free flow of resources. This loosening of regulations has significantly stimulated market vitality. By 2018, the rural land transfer rate reached 36.79%, an increase of 21.18% compared to before the policy was implemented. Meanwhile, the number of migrant workers returning to rural areas to start businesses reached 7.8 million, a 62.5% increase compared to before the policy was implemented, effectively activating the endogenous driving force of rural development. On this basis, the policy has focused on increasing fiscal transfer payments to address gaps in rural public services. In the field of education, in 2018, the per-student educational expenditure for rural middle school students was 13,912 yuan, an increase of 41.2% compared to before the policy was implemented. Meanwhile, the rate of rural participation in the new rural cooperative medical insurance reached 99.45%, an increase of 0.56% compared to before the policy was implemented, narrowing the gap in basic public services between urban and rural areas and providing solid livelihood guarantees for urban-rural integration. Furthermore, the policy has focused on promoting the deep integration of urban and rural industries. By cultivating modern agricultural industrial parks, building characteristic towns, and other platforms, it has guided urban capital and technology to flow into rural areas, integrating them with rural resource endowments and ecological advantages. By 2018, the added value of rural secondary and tertiary industries accounted for 92.8% of GDP, an increase of 2.3% compared to before the policy was implemented, gradually forming a new pattern of linked industrial development between urban and rural areas. Compared to existing studies, this research not only validates the role of policies proposed by Ma in promoting urban-rural integration, but also reveals that the policies mainly promote rapid urban-rural integration through a four-dimensional mechanism of "institutional breaking-resource activation-service balancing-industry linkage."

However, after entering 2019, the outbreak of the COVID-19 pandemic became a key obstacle to the slow development of urban-rural integration. In order to prevent the spread of the virus, the government implemented strict transportation controls and restrictions on interregional movement, which temporarily hindered the normal flow of production factors between urban and rural areas. By 2020, the number of migrant workers leaving for work was only 169.59 million, a decrease of 2.67% compared to the previous year. Meanwhile, the agricultural product logistics system was inefficient, leading to a disconnection between regional production and sales of fresh agricultural products. In some major agricultural production counties, the unsold rate exceeded 25%, significantly weakening the urban-rural economic cycle and causing a temporary decline in rural household incomes. Against this backdrop, emerging rural businesses relying on labor mobility and scene-based consumption were particularly hard-hit. By 2020, rural tourism revenue amounted to 600 billion yuan, a 66.9% decrease compared to the previous year. A large number of small businesses and individual operators, dependent on cultural and tourism services, faced existential crises. Compared to existing studies, this research not only identifies the pandemic as an exogenous shock, as emphasized by Li, but also further reveals that its impact mainly slowed down the urban-rural integration process through three core aspects: "disrupting the flow of resources—weakening the urban-rural economic cycle—impacting the rural industrial ecosystem."

Economic structural adjustment is the key driving force behind the "fluctuating upward" trend in urban-rural integration development. Alongside market demand contraction and the widespread adoption of digital technologies, traditional heavy industries and the energy sector are generally facing declining efficiency and shrinking employment, which has, to some extent, exacerbated the income gap between urban and rural areas, creating phase-specific pressures on the integration process. At the same time, emerging business models such as leisure agriculture and rural tourism have flourished, not only driving the continued expansion of e-commerce for agricultural products but also accelerating the influx of social capital into new rural sectors. These new industries effectively absorb the production factors released by the transformation of traditional sectors, creating a large number of new employment opportunities in rural areas, significantly expanding farmers' income channels, and injecting sustained and powerful endogenous momentum into urban-rural integration. In this transformation process, there is a clear dynamic tug-of-war between the decline of traditional industries and the rise of emerging industries: the short-term pain caused by the adjustment of traditional sectors intertwines with the long-term benefits brought by the expansion of the new economy. However, as the growth potential and driving effects of the new driving forces continue to strengthen, their upward momentum generally surpasses the downward pressure brought by the decline of traditional sectors, thus driving the overall "fluctuating upward" development trend of national urban-rural integration. Compared to existing research, this paper not only verifies the profound impact of economic structural adjustments on urban-rural integration development, as proposed by Xu, but also deepens the understanding of the structural causes behind the "fluctuating upward" trend in urban-rural integration from the perspective of the transition between old and new driving forces.

**Analysis of spatial distribution characteristics**

The urban-rural integration development in China shows distinct spatial heterogeneity, forming a diverse development pattern where the eastern coastal region leads, the central region rises rapidly, and the western region has great potential. This spatial structure is closely related to the basic conditions and development paths of different regions. The eastern coastal regions, benefiting from superior geographical locations, have actively developed an open economy, gradually building a solid economic foundation, attracting large amounts of capital and high-end industries, and effectively promoting the early development of urban-rural integration through industrial gradient transfer mechanisms. Taking Zhejiang Province as an example, this province is located on the southeast coast and is home to the Ningbo-Zhoushan Port, the largest cargo throughput port in the world. Its unique geographical advantages provide a solid foundation for the development of an open economy. Leveraging this condition, Zhejiang has vigorously developed port industries and cross-border e-commerce, gradually building a strong economic base. By 2022, Zhejiang’s per capita GDP reached 118,496 yuan, ranking fourth among all provinces and regions in China. On this basis, the province has attracted a large amount of capital and high-end industries by improving infrastructure and continuously optimizing the business environment. However, as the costs of land, labor, and other factors in the central cities continue to rise, many high-value-added industries such as export processing and smart manufacturing have gradually spread to counties and rural areas with larger development spaces and lower overall costs. This process has significantly driven industrial upgrading and expanded employment opportunities in rural areas, effectively narrowing the urban-rural development gap. By 2022, the urban-rural income ratio in Zhejiang had dropped to 1.90, significantly lower than the national average, making it one of the provinces with the most significant achievements in urban-rural coordinated development. As pointed out by Wang's research, the economic development level plays a key role in urban-rural integration. However, this study further emphasizes that urban-rural integration not only depends on the overall economic level but is also closely related to the restructuring of industrial space, actively promoting urban-rural integration towards a higher level of development.

The central region, benefiting from its abundant labor resources, has effectively aligned with the market demands of the eastern coastal regions. An increasing number of labor-intensive industries such as textiles and apparel, electronics, and home appliance manufacturing have chosen to relocate to the central region, where factors like land and labor costs have a comparative advantage. In this process, large amounts of capital, technology, and management expertise have continuously flowed into the central region through direct investment, industrial transfer, and regional cooperation, not only driving the transformation and upgrading of local traditional industries but also accelerating the agglomeration of emerging industries. It is particularly noteworthy that these industrial transfers have not only remained at the city level but have shown significant "county-oriented" characteristics. Taking Henan Province as an example, the province has actively taken on industrial transfer from the Yangtze River Delta region by building modern industrial clusters, creating several specialized industrial clusters at the county level. For example, the textile and apparel industrial cluster in Taikang County, Zhoukou City, not only attracted well-known enterprises from the eastern region but also extended the industrial chain to rural areas through a "leading enterprise + rural workshop" model, directly providing employment and income growth for nearly 100,000 rural laborers. The smart sensor industrial cluster formed in Nanyang City, in addition to setting up core research and manufacturing links around the city, has also dispersed supporting links such as packaging, logistics, and basic component production to surrounding towns, promoting the deep integration and collaborative development of urban and rural industrial chains. As pointed out by Ma's research, industrial transfer has injected strong momentum into urban-rural integration. However, this study further emphasizes that the deep embedding of industries at the county and rural levels not only promotes the reallocation of production factors between urban and rural areas but also significantly drives urban-rural integration towards higher quality development through job creation and industrial chain collaboration.

In contrast, the western region faces more prominent urban-rural dual structure issues due to its inland location and complex ecological environment. However, with the continuous support of national strategies such as the Western Development Program, poverty alleviation, and rural revitalization, the region has made significant progress in breaking through the bottleneck of urban-rural integration. Taking Sichuan Province as an example, its "characteristic agriculture + rural tourism" dual-driven model in the Qinba Mountains and Liangshan Yi Autonomous Prefecture has not only activated rural industries but has also become an important mechanism for connecting urban and rural areas and promoting integration. On one hand, by developing specialty industries such as highland agriculture and ecological breeding, and leveraging ethnic culture and ecological resources to promote rural tourism, rural areas have gradually established high-value-added industrial systems, strengthening their connections with urban consumer markets. On the other hand, the opening of e-commerce channels has not only achieved the smooth flow of both "agricultural products going up" and "industrial products going down," but has also profoundly transformed the traditional urban-rural exchange model, promoting the more fair and efficient flow of resources and elements between urban and rural areas. These measures have not only promoted income growth for farmers and diversified rural economies but have also fundamentally reshaped urban-rural relations. Rural areas are no longer a simple resource exporter or a passive recipient of development but are gradually growing into development subjects that complement urban functions and share value. In this process, characteristic industries have become the link for urban-rural industrial collaboration, e-commerce platforms have become bridges for connecting urban and rural markets, and policy guidance has provided a solid guarantee for institutional integration between urban and rural areas. As pointed out by Wang's research, policy support plays a key role in driving urban-rural integration. However, this study further emphasizes that the integration of urban and rural areas in the western region not only depends on external resource inputs but, more fundamentally, on the construction of endogenous driving mechanisms. Only by fully unleashing the local development potential of rural areas can urban-rural integration move towards a more sustainable direction.

**Analysis of driving factors**

The development of urban-rural integration in China is influenced by multiple factors, including infrastructure, economic development, public services, social security, and the ecological environment. Among these, broadband internet access, per capita disposable income, education expenditure, unemployment insurance coverage, and green coverage rate have played key roles in promoting this development.

Specifically, broadband internet access is the cornerstone of urban-rural information resource sharing, directly impacting the speed of information transmission, knowledge acquisition, and cultural exchange. However, due to underdeveloped infrastructure, the broadband access rate in rural areas is generally lower than in urban areas, leading to a significant digital divide. Therefore, reducing the gap in broadband access between urban and rural areas is crucial for achieving information sharing and further promoting urban-rural integration.

Per capita disposable income, as an important indicator of economic development, directly reflects the consumption capacity of urban and rural residents. However, due to the simple industrial structure and backward production technology in rural areas, per capita disposable income is generally lower than in urban areas, which continues to widen the income gap between urban and rural areas. This income disparity not only lowers the consumption capacity of rural residents but also hinders the overall economic development of rural areas. Therefore, narrowing the gap in per capita disposable income between urban and rural areas is of paramount importance for improving the level of economic development and promoting urban-rural integration.

Education expenditure is a key means of improving the quality of human capital in urban and rural areas. However, rural areas currently face a shortage of educational investment, primarily due to the low income levels of rural families, which makes it difficult for them to bear high educational costs. This disparity in educational resources not only exacerbates the inequality in educational opportunities between urban and rural residents but also severely hinders the overall improvement of human capital quality in rural areas. Therefore, reducing the gap in education expenditure between urban and rural areas is crucial for enhancing human capital quality and promoting urban-rural integration.

Unemployment insurance coverage is an important indicator of the completeness of the social security system in urban and rural areas. However, due to inadequate institutional design, the unemployment insurance coverage rate in rural areas is generally lower than in urban areas. This disparity not only leaves rural residents inadequately protected against the risk of unemployment but also limits the economic development of rural areas. Therefore, narrowing the gap in unemployment insurance coverage between urban and rural residents is crucial for improving the social security system and promoting urban-rural integration.

Green coverage rate is an important indicator of regional ecological environmental quality. However, due to economic advantages and policy support, urban areas generally have a higher green coverage rate than rural areas. This disparity not only results in unequal access to green ecological benefits for urban and rural residents but also hinders the improvement of the ecological environment and quality of life in rural areas. Therefore, narrowing the gap in green coverage rate between urban and rural areas is crucial for improving regional ecological environmental quality and promoting urban-rural integration.

We greatly appreciate the opportunity to revise and resubmit our manuscript. All changes have been incorporated into the revised version, and we believe the manuscript has been significantly improved thanks to your and the reviewers’ insightful comments. We look forward to your positive response. Thank you once again for your time and guidance.

Sincerely,

Wei Wang
